# Supplementary material for: Promoters maintain their relative activity levels under different growth conditions
Source: Mol Syst Biol. 2013 Oct 29;9:701. doi: 10.1038/msb.2013.59 (PMC3817408; doi:10.1038/msb.2013.59)
Supplement: Supplementary information — Supplementary Figures S1–19 [file msb201359-s1.pdf]

Promoters maintain their relative activity levels under different growth conditions

Supplementary Information

Contents

1. Supplementary results and discussion 2
1.1 Selection of genes for promoter library. 2
1.2 Analysis of mRNA and protein datasets. 3
1.1.1 Promoter activity is highly correlated with mRNA and protein levels 3
1.1.2 Proportional scaling is largely preserved for both mRNA and protein levels 4
1.3 Discussion of experimental system 5
1.3.1 Fluorescence as a measure for promoter activity 5
1.3.2 Genomic location 7
1.3.3 Advantages and assessment of accuracy of the experimental system: 8
1.4 Models for global effects on expression across conditions. 9
1.5 Variance explained by linear scaling 10
1.6 Enrichment analysis 11
1.7 Accurate prediction of promoter activities from several representative promoters 13
1.8 Expansions on the passive resource allocation model 14
2. Supplementary methods- S. cerevisiae 16
2.1 Library design and construction. 16
2.2 Promoter activity measurements. 16
2.3 Growth conditions. 16
2.4 Computing promoter activity levels. 16
2.5 Estimating the detection level of our experimental system. 18
2.6 Experimental Variability. 18
2.7 Global scaling factors and error model. 18
2.8 Quantitative PCR analysis. 19
2.9 Functional annotation and enrichment analysis. 20
2.10 Clustering promoter activities. 20
2.11 Variance explained by clustering. 21
2.12 Predicting promoter activity levels. 21
3. Supplementary methods- E. coli 23
3.1 Growth conditions. 23
3.2 Robotic assay for genome-wide promoter activity data. 23
3.3 Computing promoter activity levels, detection level, experimental variability and error model 23
4.Supplementary figures 25
References 50

1. Supplementary results and discussion

1.1 Selection of genes for promoter library.

Our study encompasses 1820 (3/4) of all *E.coli* promoters and 859 (1/6) of all *S.cerevisiae* promoters. The *E.coli* dataset was previously described (Zaslaver *et al*, 2006) and was shown to be highly representative of both genome and proteome. The yeast promoters were chosen to include several complete groups of genes, in addition to representatives of other groups. Thus, our library includes nearly all known ribosomal proteins (110 promoters), transcription factors (157 promoters) and central metabolism enzymes (312 promoters). The remaining 280 promoters were chosen to represent various GO categories: cytoskeleton, cell wall, DNA processing, chromatin, mRNA processing and export, chaperons, cell cycle genes, signaling pathways, secretory pathways, mitochondrial proteins, vacuolar proteins, degradation pathways etc. In total, our library covers ~1/6 of the *S. cerevisiae* genome.

We performed several tests to gauge the degree of representation of our library. First, we examined the distribution of our promoters with respect to dominance of either TFIID or SAGA transcription initiation complexes, as previously determined (Huisinga & Pugh, 2004). We found that our set of promoters is representative of the genomic distribution, with 78% TFIID-dominated promoters and 13% SAGA-dominated promoters (similar to the genomic percentages of 82% and 10%, respectively, **Table S1** (Huisinga & Pugh, 2004)). This indicates that our set of promoters is representative of the two main transcription regulation strategies, not enriching for neither housekeeping nor stress-related genes.

Second, we examined our promoters with respect to their architecture. We classified the promoters of all yeast non-dubious open reading frames and known non-coding RNAs according to their promoter type. Promoters which support bidirectional transcription (a head to head orientation) were classified as Divergent, and promoters that support unidirectional transcription (a tail to head orientation) were classified as Unique. We found that our set of promoters is representative of the genomic distribution, with 50% divergent promoters and 50% unique promoters.

Third, we examined our promoters with respect to the two characteristic promoter architectures, OPN and DPN (Tirosh & Barkai, 2008). We found that out of our 859 promoters, 162 were designated with prominent promoter architectures. Of these, 65 were designated as DPN and 97 as OPN, indicating that our set of promoters is well representative of promoter architectures, not enriching for neither closed nor open forms.

Fourth, we examined protein expression levels of our set of genes in a curated dataset of protein abundances integrated from 5 different datasets (Wang *et al*, 2012). We found that the combined expression of our selected genes encompasses ~60% of the protein mass expressed in rich media. We note that while this subset is enriched for highly active promoters, and therefore may not accurately represent weak promoters, it is highly representative of the proteome, and accounts for much of the cellular activity in standard growth conditions.

Fifth, we repeated our entire analysis, excluding ribosomal promoters overrepresented in our datasets, and our results were hardly affected, both qualitatively and quantitatively (**Fig. S10**).

1.2 Analysis of mRNA and protein datasets.

We explored whether our findings regarding proportional scaling of promoter activities also apply for mRNA and protein abundance, as these include additional layers of regulation. To this end we performed several analyses:

1.1.1 Promoter activity is highly correlated with mRNA and protein levels

We first compared our measurements in synthetic complete media with glucose to a variety of existing datasets that examined genome-wide mRNA and protein levels under rich conditions. We compared our promoter activity values to 3 different DNA microarray studies (Holstege *et al*, 1998; Shalem *et al*, 2008; Lipson *et al*, 2009), 3 different RNA-seq studies (Nagalakshmi *et al*, 2008; Lipson *et al*, 2009; Yassour *et al*, 2009), protein abundance obtained by immuno-tagged proteins (Ghaemmaghami *et al*, 2003), fluorescently-tagged proteins (Stewart-Ornstein *et al*, 2012), mass spectrometry (de Godoy *et al*, 2008) and a curated dataset of protein abundances integrated from 5 different datasets (Wang *et al*, 2012). In all cases our promoter activity data correlated well with mRNA and protein abundance (R=0.72-0.81 and R=0.57-0.74 respectively, **Fig. S5**), suggesting that promoter activity is a major determinant of these properties.

The comparisons between all these datasets yield several interesting observations. First, we note that the correlation coefficients between our promoter activity and these datasets are similar to the correlation coefficients between these datasets themselves, supporting the validity of our data. Second, as expected, the mRNA datasets better correlate between themselves than they do with the protein datasets. Our data correlates better to mRNA levels than to protein levels, as could be expected for promoter activity data. Finally, the high correlations suggest that global

trends we derive for promoter activities should be largely preserved for both mRNA and protein levels. We test this in the following section.

1.1.2 Proportional scaling is largely preserved for both mRNA and protein levels

To test whether our findings regarding proportional scaling of promoter activities also apply for mRNA and protein abundance, we searched the literature for genome-wide studies performed in several growth conditions. We note that these datasets are not ideal for the detection of proportional scaling due to the following reasons:

1. While microarrays and RNA sequencing are very useful at delineating global profiles, they are known to have considerable noise at the single gene level (Marshall, 2004; Frantz, 2005). These stem from technical challenges such as gene-specific biases in hybridization, PCR, differences in labeled material, dye-specific biases and location on the array (Oshlack & Wakefield, 2009; Balázsi & Oltvai, 2007). For these reasons, a scatter that compares genes between conditions will generally form a 'cloud' rather than a tight line that would indicate a proportional response **(Fig. S3)**.
2. Most genome-wide technologies employ various normalization procedures to counteract the abovementioned non-biological variation, such as analysis of equimolar amounts of cDNA after cell lysis and RNA extraction and various in-silico normalizations (Churchill, 2002; Tang *et al*, 2007; Bammler *et al*, 2005; Bakel & Holstege, 2008a; Sun *et al*, 2012). These contribute to the obliteration of global proportional scaling.
3. Most of these experiments do not include biological replicates, making it impossible for us to separate the experimental noise from the actual change in gene expression and to tease apart global and specific responders, as we did in this study for both the *S. cerevisiae* and *E. coli* data (Methods).

For the reasons above, we took a complementary approach. We reasoned that if the genes that display proportional scaling in their promoter activity also display it in other levels of regulation, then we would expect that when we examine the ratio of these genes between conditions, it will be relatively constant. That is, if proportional scaling is preserved, the expression ratio between two conditions of genes that belong to the same cluster in our analysis should have low variability. However, due to the lack of replicates in these experiments we do not have an estimate of the experimental noise and absolute measure of what 'low variability' is. Even so, we can predict that if our principle holds, this variability should be lower than the variability between genes from different clusters that scale with different scaling factors. We note that this analysis depends on our

clustering, which was derived from a defined set of environmental conditions, and therefore it can be performed only on datasets that examined similar conditions.

We thus compared the intra-cluster variability to the inter-cluster variability of genes that were part of this study in 8 datasets, including microarrays (Gasch *et al*, 2000; Lai *et al*, 2005; Chechik *et al*, 2008; O'Rourke & Herskowitz, 2002; Brauer *et al*, 2008), RNA-seq (Tirosh *et al*, 2011), fluorescent protein-fusion strains (Breker *et al*, 2013) and mass spectrometry (Costenoble *et al*, 2011). We asked to what extent genes within the global cluster preserve proportionality between conditions by examining whether the ratio of these genes between the conditions was less variable than the ratio between the other genes between the same conditions. Indeed, for all examined conditions in all examined datasets, we found that the intra-cluster variability was much smaller than the inter-cluster variability (**Fig. S11**), indicating that the proportionality we observed for promoter activity is largely preserved for mRNA and protein levels.

1.3 Discussion of experimental system

Our experimental system is based on fusion of promoters upstream of a fluorescent reporter and enables non-invasive tracking of live cells across time, as described in detail in the main text and experimental procedures. This is an established approach, and promoter-reporter constructs using enzymatic reporters such as beta-galactosidase or luciferase have been used successfully in biological research since the 1980s (Bronstein *et al*, 1994) and have contributed much to our understanding of promoter architecture and gene regulation. More recently, variants of this approach using fluorescent reporters were successfully applied by our lab and by others to reveal ordered activation of genes in various pathways in bacteria (Kalir *et al*, 2001; Zaslaver *et al*, 2004) and to generate libraries of native and synthetic promoters in bacteria (Cox *et al*, 2007) and yeast (Ligr *et al*, 2006; Murphy *et al*, 2007; Gertz *et al*, 2009; Raveh-Sadka *et al*, 2012; Sharon *et al*, 2012; Zeevi *et al*, 2011; Newman *et al*, 2006), which provided much insight into the rules that underlie combinatorial cis-regulation. We hereby discuss some of the advantages and limitations of our system and provide several reasons and validations for its accuracy and sensitivity.

1.3.1 Fluorescence as a measure for promoter activity

An important feature of our experimental system is that we rely on a fluorescent protein as a reporter for promoter activity. The use of fluorescence has several well-established advantages, such as high sensitivity and measurement resolution (**Fig. S1-**

3) and the ability to perform the measurements in-vivo. However, the use of fluorescence may also be regarded as a limitation of the system as the reported values encompass changes in both transcription and translation. We note that this is true for all reporter assays, including immuno-tagged proteins, luciferase and beta-galactosidase assays, which have nevertheless contributed highly to our understanding of the regulation of expression (Bronstein *et al*, 1994). We believe the advantages of fluorescence reporters outweigh their disadvantages. Yet, since we realize that this is the shortcoming of our experimental system, we have performed several validations with respect to this issue:

1. All the tested promoters drive the expression of the same protein (YFP) with the same 3'-UTR. Thus, aside from the 5'-UTR, the transcript produced from all promoters is identical. It is thus reasonable to assume that post transcriptional and post translational regulation is highly similar for all of our strains, and consequently, that differences in the measured promoter activity for different genes are attributable to differences driven by the different promoter sequences. Naturally, between conditions all promoters will also be affected by changes in global translational parameters, yet these effects should be similar for all tested promoters.
2. We compared our promoter activities to quantitative real-time PCR measurements of 18 selected strains in two growth conditions. The high correlation obtained between mRNA and YFP levels (R = 0.99 and R = 0.98, **Fig. S4A-B**) and the recapitulation of our results at the mRNA level (**Fig. S4C-D**) confirm that YFP protein levels are an accurate proxy for the corresponding mRNA levels.
3. We compared our promoter activity values to three DNA microarray studies (Holstege *et al*, 1998; Shalem *et al*, 2008; Lipson *et al*, 2009), three RNA-seq studies (Nagalakshmi *et al*, 2008; Lipson *et al*, 2009; Yassour *et al*, 2009), protein abundance obtained by immuno-tagged proteins (Ghaemmaghami *et al*, 2003), fluorescently-tagged proteins (Stewart-Ornstein *et al*, 2012), mass spectrometry (de Godoy *et al*, 2008), and a curated dataset of protein abundances integrated from five different datasets (Wang *et al*, 2012). We found that the correlation coefficients between our promoter activity and these datasets are similar to the correlation coefficients between these datasets themselves, supporting the validity of our data. In addition, our data is better correlated with the mRNA datasets (R=0.72-0.81, **Fig. S5**) than with the protein datasets (R=0.57-0.74 **Fig. S5**), as could be expected from promoter activity.

4. We analyzed eight datasets of mRNA and protein levels including microarrays (Gasch *et al*, 2000; Lai *et al*, 2005; Chechik *et al*, 2008; O'Rourke & Herskowitz, 2002; Brauer *et al*, 2008), RNA-seq (Tirosh *et al*, 2011), fluorescent protein-fusions (Breker *et al*, 2013) and mass spectrometry (Costenoble *et al*, 2011). We found that our results were largely recapitulated by these datasets, as detailed in the section above (**Fig. S11**).

For all these reasons, we believe that the values we report are highly indicative of the actual promoter activities.

1.3.2 Genomic location

Another feature of our experimental system is that all constructs are integrated at the same locus in the yeast genome. Similar to the use of fluorescence, this trait of our experimental system has both advantages and disadvantage. One concern is that the epigenetic regulation of all constructs is likely to be identical, and therefore the reported values will not be indicative for the promoters in their native contexts. To assess whether this is indeed a major concern, we compared our results to 10 distinct datasets of mRNA and protein abundance that were measured in the native genomic locations, as detailed above. The high correlations obtained (R=0.57-0.81, which is similar to the correlations between these datasets **Fig. S5**), and the recapitulation of our results in several of these datasets (**Fig. S11**), reinforce our values to be adequate proxies for these quantities, despite the non-physiologic location of the promoters in our experimental system. In addition, we note that our results also hold for the prokaryote *E.coli*, in which genomic location is presumably a less significant factor due to the absence of chromatin.

An advantage of our experimental system is that it isolates the effect of the promoter alone, without effects of genomic context, distinct coding sequences and various post-transcriptional modifications. All the differences in expression between different strains in the library can be attributed to the ~400bp inserted upstream to the YFP. This allows us to obtain a clean signal for promoter activity, which is required for mathematical modeling of the promoter contribution to the response to different conditions. Thus, it appears that the fixed location of the promoters in our experimental system does not have a major effect on the outcome of the study and is beneficial in the attribution of the observed phenomenon to the promoter sequence. Repeating our experiments with the promoters at their native location could be informative of how much of the transcriptional signal is attributed to the promoter versus the genomic location, which is in itself an interesting and unresolved question.

1.3.3 Advantages and assessment of accuracy of the experimental system:

We hereby provide several reasons and validations for the accuracy and sensitivity of this system:

1. The choice of fluorophore: We chose YFP over GFP, which was used in protein-fusion libraries (Newman *et al*, 2006), since yeast cells autofluoresce much less at this wavelength, thereby increasing our measurement sensitivity (*35,* Fig. S1).
2. YFP stability: it has been shown that YFP is stable and long-lived. Whereas this property restricts the ability to interrogate dynamically changing systems (Mateus & Avery, 2000; Houser *et al*, 2012), it is ideal for interrogation of steady state growth as slow turnover rate ensures that the difference in YFP levels across time provides a direct measure of the amount of YFP produced.
3. In addition to the YFP, we inserted a red fluorophore (mCherry) which serves as an internal control for the reliability of the strain construction process and experimental variability. When constructing a fluorescence-based library, even when it is done robotically, each strain undergoes separate transformation, growth and measurement. It is therefore critical to have a control showing that in the process the different strains in the library did not acquire any mutations that generally affect transcription in the cell, causing the promoter activity calculated from the YFP to be unreliable. Indeed all strains in the library exhibit highly similar growth curves and mCherry levels (**Fig. 1**).
4. The construction of the strains and experimental procedures were subjected to several tests including: sequencing of promoter insert, similarity in growth curves and similarity in mCherry expression.
5. Our system requires neither interventions nor normalizations, as we non-invasively track live cells over time with high temporal resolution, enabling the extraction of robust data.
6. We validated that the YFP levels of independent clones of the same promoter sequence are indistinguishable from those of replicate measurements of the same clone, indicating that our library construction procedure does not introduce mutations that have global effects on transcription or translation (*35,* **Fig. S4**).
7. For 60% of strains and conditions, measurements were performed in biological replicates (up to 6 measurements per strain), which were carried out on different days and included all stages of the experiment, starting from the frozen stock. This allowed us to reliably assess our detection level and experimental noise. We

note that we report low coefficient of variation values at the single gene level, ranging from 0.05 for highly active promoters to 0.36 for promoters with very low activity, lower than those obtained with other methods such as microarrays, RNA-sequencing and mass-spectrometry (Methods, **Fig. S1-3**).

8. The accuracy of our system is supported when we examine the GO annotations of the genes in our proportional clusters (**Table S6**). We find that in nearly all cases members of the same pathway or complex cluster together, resulting in near maximal enrichment values.
9. Quantitative PCR elaborated above indicates that our measurements of promoter activities correlate well with mRNA levels (R = 0.99 and R = 0.98, **Fig. S4A-B**).
10. Good correlation of our dataset to existing mRNA and protein datasets elaborated above (**Fig. S5**) and the recapitulation of our main finding in these datasets (**Fig. S11**) reinforce the validity of our data.
11. The repetition of the phenomenon of proportional scaling in the independent dataset from *E.coli* provides further support for the conclusions extracted from our data (**Fig. 7,S12-S17**).

1.4 Models for global effects on expression across conditions.

In our study, we found that promoter activities change between conditions and that this change is captured by a linear function with a scaling factor that according to our model compensates for the changes in growth rate and magnitude of the specific response. This proportional response agrees with theories of the Copenhagen school, which proposed that the expression of non-regulated genes should scale proportionally (Maaloe, 1969; John L. Ingraham, Ole Maaløe, 1983). Here we discuss plausible alternative modes of regulation that would lead to different findings:

- **Static response**: Non-regulated promoters could be static between conditions, with no expression change whatsoever. Although this model may seem obsolete, this assumption actually underlies most current forms of analysis of high throughput data, including microarray and sequencing analysis (Bakel & Holstege, 2008b).
- **Highly variable response**: Promoters, both regulated and non-regulated, could display high variability between conditions, with no preservation of their relative values. This model resonates with the current paradigm that gene expression is the result of a highly complex combinatorial process, whereby each gene is separately controlled by a distinct set of both global and specific transcription

factors (Reményi *et al*, 2004). Across conditions, this model predicts changes in the production of each of these factors, which in turn would lead to differential changes in expression of their targets. The outcome of this would appear as a highly variable response across conditions, with little or no preservation of stoichiometry between different genes. Graphically, when comparing between two conditions, this will manifest as a 'cloud', rather than a tight line.

- **Linear response with a different scaling factor**: Non-regulated promoters could display proportional changes between conditions with various optional scaling factors. One plausible hypothesis discussed in the main text is that such a global scaling factor will quantitatively compensate for the changes in growth rate. This model requires cells to possess a mechanism for coordinating promoter activity and doubling time. In principle, this could be achieved if cells control their growth rate based on the concentration of some non-specifically regulated 'counter protein'. Higher concentration of that protein would lead to faster growth rates which in turn would decrease the concentration. This model will entail the global scaling factor to be proportional to the growth rate. This model is compelling as it entails that per doubling time, unregulated promoters will preserve their concentration, which could be beneficial for the robustness of the cell.
- **Non-linear response**: Another model is that global changes in parameters will have a differential effect on different promoters. Thus, gene expression changes between conditions resulting from global regulation may obey a non-linear mathematical function. It is easy to suggest molecular mechanisms that will exert such differential responses of the promoters. For example, if the determining factor was RNA polymerase, then we would have expected that promoters with different architectures and different affinities for the polymerase will be affected differentially.

1.5 Variance explained by linear scaling

The adherence of promoter activities to scale lines may seem at first somewhat surprising, in light of the prevailing paradigm that gene expression is the result of a highly complex combinatorial process, whereby each gene is separately controlled by a distinct set of both global and specific transcription factors (Reményi *et al*, 2004). We

believe our results do not contradict this paradigm, but rather allow one to assess the relative contribution of such different factors.

We find that across all conditions, partitioning promoters into 6 clusters allowed us to account for 97% of the variability in promoter activities across conditions over the entire dataset. This analysis can be repeated separately for each condition and for each cluster (Methods). We find that for all conditions, the use of only 6 scaling lines explains 72%-98% of the variability in the dataset. The existence of residual variability represents the fact that promoter activities do not adhere perfectly to their respective scale line, but are rather distributed around it (**Fig. 3C-E,G, Fig. S7**). This distribution indicates that in addition to the linear scaling, shared by different group members, there exist some gene-specific changes in activity. This can be caused by different sensitivities and non-linearities in the response that different group members have to changes in global and specific transcription factors. However, the fact that such high percentages of the response are quantitatively explained by only six numbers, suggest that these gene-specific changes are small compared with the changes shared across functionally-related clusters and the global changes across conditions. Altogether, our results imply that despite being highly combinatorial, gene expression changes across conditions may actually be quantitatively structured and simple, since most of this change can be captured by only a few scale lines.

Adding conditions to our study would help to identify more condition-specific promoters. As more conditions are added, it would be interesting to see how many more numbers are required to fully understand an organism's expression programs. It would be interesting to see if there is an upper limit on the number of scaling factors needed to fully predict the expression program in a new condition. We hypothesize that this number is possibly similar to the number of specific regulators in the organism (on the order of 250 in *E. coli* and *S. cerevisiae*).

1.6 Enrichment analysis

To assess our clustering, we examined them in terms of biological function. First, we tested their enrichment in functional annotations from Gene Ontology (GO) (Methods). Next, we asked whether our clusters grouped together promoters that are regulated by similar mechanisms. To this end, we examined how promoters of transcription factors (TFs) were distributed across the different clusters, since changes in the expression of many TFs were shown to correlate with changes in the expression of their targets (Segal *et al*, 2003; Pe'er *et al*, 2002). Our library allows a global

examination of this idea, since it includes promoters for most of the known yeast transcription factors (Badis *et al*, 2008; Zhu *et al*, 2009). Finally, we examined the promoters in terms of their promoter architectures (OPN/DPN (Tirosh & Barkai, 2008), promoter types (divergent/unique, based on (Saccharomyces Genome Database)) and transcription regulation strategies (SAGA-dominated/TFIID-dominated (Huisinga & Pugh, 2004)) (Methods).

Notably, we found significant enrichments across all six clusters, in good correspondence with our understanding of the tested conditions (**Fig. 4, Tables S6,S7**). As detailed in the main text, the first cluster contains most genes (77%) and most TFs (85%), from various GO families. It is enriched for constitutive, TATA-less, TFIID-regulated promoters with an open chromatin architecture. While the expression of some of these genes (e.g., ribosomal protein genes) was previously shown to be correlated with the growth rate (Regenberg *et al*, 2006; Castrillo *et al*, 2007; Brauer *et al*, 2008), others (e.g., GAPD and actin) are considered classical house-keeping genes that are constitutively expressed. Our measurements indicate that the activity of all of these promoters scales together across all 10 tested conditions.

The other clusters exhibit condition-specific responses in a subset of the tested conditions and are highly enriched for families of genes that are known to respond to these conditions and their known regulators (**Fig. 4, Tables S6,S7**, supp. material 1.6). Clusters 2 and 5 represent two different branches of respiration and are highly upregulated in strictly aerobic conditions (ethanol and glycerol) and mildly upregulated in partially aerobic conditions (galactose and galactose lacking amino acids). Cluster 3 is upregulated in osmotic stress conditions (NaCl) and is enriched for trehalose and glycoside metabolism, known to participate in alleviation of this stress. Cluster 4 is upregulated in conditions lacking amino acids (with either glucose or galactose as carbon sources) and is enriched for genes of amino acid biosynthesis pathways. As expected, clusters 2-4 are also enriched for SAGA-dominated promoters. Finally, cluster 6 is upregulated in conditions in which galactose serves as the carbon source, and it is comprised almost solely from promoters of genes from the galactose assimilation pathways. Thus, these clusters may represent complete regulatory units that are coregulated across conditions in a manner that largely preserves their internal stoichiometry.

An important implication of our clustering results is that proportional scaling of promoter activities transcends the usual partition of promoters to housekeeping

/condition-specific, open/closed, TFIID/SAGA-dominated. When examining the matrix of scaling factors of clusters across conditions (Table S5), it is clear that between most conditions, most clusters are not differentially regulated and their scaling factor coincides with the global scaling factor. This is consistent with the observation that cluster 1 contains condition-specific promoters that are not differentially regulated across our tested set of conditions (e.g. ER-stress associated proteins), and therefore scale according to the global scaling factors across the entire dataset. These observations hint that the mechanisms responsible for global proportional scaling are not unique to a limited set of genes, promoter architecture or transcription regulation strategies. We find that both growth-related promoters (e.g. ribosomal) and stress-related promoters (e.g. respiration-related metabolism) exhibit global proportional scaling when not differentially regulated. Accordingly, both TFIID-dominated and SAGA-dominated promoters exhibit global proportional scaling when not differentially regulated. Thus, global proportional scaling of promoter activities is probably the result of a basic mechanism, shared across all promoter classes and architectures.

1.7 Accurate prediction of promoter activities from several representative promoters

The clustering described in the main text implies that in any given condition, the activity levels of all promoters can be accurately described by knowing the partition of promoters into clusters, the relative activity levels of promoters within each cluster, and the clusters' scaling factors in the given condition. Both the clustering and relative activity levels of promoters in each cluster can be obtained from promoter activity measurements in some set of conditions. The scaling factors for any new condition can be obtained by measuring the activity level of only a few representative promoters from each cluster in that new condition. Thus, we hypothesized that given promoter activity measurements in some set of conditions, we should be able to accurately predict all of the promoter activities in a new condition by measuring only a few representative promoters. To test this hypothesis, we used a cross-validation scheme, whereby for each tested condition, we clustered the promoters based on all other conditions, and predicted all of the promoter activity levels of the tested condition using the activity levels of only a few predefined representative promoters (Methods). Indeed, using only 10 promoters, we obtained highly accurate activity level predictions, whereby in every condition, the predictions explain over 85% of the variance of the activities of at least 98% of the promoters (**Fig. 5, S9**).

This ability to derive accurate predictions results from the variety of specific responses represented in our dataset, and from our finding that most promoters preserve their relative activity levels across conditions. Since we can only accurately predict conditions that are similar to conditions that were already measured or to combinations of such conditions, we fail to predict specific responses that were not contained in the original training dataset (**Fig. 5C**, blue boxes). However, these failures are highly informative, since promoters for which our predictions deviate the most from their measured activity levels suggest the existence of new clusters and regulatory pathways that were not activated in the original dataset.

1.8 Expansions on the passive resource allocation model

We suggest a passive resource allocation model to explain the values of the observed global scaling factors. We posit that in every doubling the total number of molecules produced from all promoters together is preserved. Such preservation of total promoter activity is expected without the need for an elaborate regulation mechanism if the total fraction of proteins in the biomass (measured as OD) is close to constant across conditions (Maaloe, 1969; John L. Ingraham, Ole Maaløe, 1983). In line with earlier models discussing differential allocation of resources between conditions (Ehrenberg & Kurland, 1984; Koch, 1988; Scott *et al*, 2010; Zaslaver *et al*, 2009; Molenaar *et al*, 2009), the overall promoter activity can be thought of as a fixed resource available to the cell per doubling time, but that this resource is differentially partitioned between the condition-specific genes, and the globally responding genes, where the exact partition is determined by the varying magnitudes of the specific response required in each condition. Thus, the value of the global scaling factor will accommodate both changes in growth rate and the magnitude of the specific response in each condition.

Since all ribosomal subunit promoters in our data belong to the global cluster, this model is consistent with previous studies in which the fraction of ribosomes in the cell was found to be correlated with growth rate (Schaechter, 1958; Maaloe, 1969; Bremer & Dennis, 1987; Zaslaver *et al*, 2009; Scott *et al*, 2010). Notably, this model entails that the concentration of most proteins per doubling time per biomass is not preserved across conditions. It is interesting to speculate how cells manage with these changes in concentration.

Despite this ability of the passive resource allocation model to account for a large fraction of the global scaling factors based on the growth rate and magnitude of the

specific response to each condition, there remains a considerable fraction of the proportional response that this simplified model does not explain. For the yeast dataset, this may partly stem from inaccuracies in our estimates of the total activity of all promoters, since our library does not include all promoters. Similarly, this simplified model does not directly account for other global properties, such as cell size and macromolecular composition, which are known to vary in different conditions along with the doubling time (Schaechter, 1958; Bremer & Dennis, 1987; Neidhardt, 1999). Nevertheless, the accuracy with which this model matches the measured global scaling factors based on the doubling times and magnitude of the specific responses suggests that these factors are likely to be major determinants of the global scaling factors.

Finally, it is interesting to consider what mechanisms are responsible for the observed proportionality and the coordination between promoter activity and growth rate. In bacteria it has been suggested that cAMP (You *et al*, 2013) ppGpp (Magnusson *et al*, 2005) and use of alternative sigma factors (Klumpp & Hwa, 2008; Zaslaver *et al*, 2009) may contribute to the differential allocation of resources to different groups of genes. In yeast, transporters, cAMP, master growth regulators (e.g. *TOR*) and different ribosomal subunits have been suggested to perform a similar task (Broach, 2012). Importantly, our current observations that proportional scaling underlies both global and specific responses hint that the mechanisms responsible for global proportional scaling are not unique to a limited set of genes, promoter architecture or transcription regulation strategy. It transcends the usual partition of promoters to housekeeping/ condition-specific, growth-regulated/stress-regulated, open/closed NFR, TFIID/SAGA-dominated. Accordingly, known growth-related sequence motifs, such as binding sites for Rap1/Ifh1/Fhl1/Sfp1/, RRPE and PAC (Hughes *et al*, 2000; Wade *et al*, 2001; Zhu *et al*, 2009; Badis *et al*, 2008) could not account for global scaling throughout all clusters. Thus, global proportional scaling of promoter activities is probably the result of a basic mechanism, shared across all promoter classes and architectures. To date the molecular mechanisms underlying proportional responses and the identity of the limiting resources, and the role that cell size, shape, and composition may play in this process remain unknown.

2. Supplementary methods- S. cerevisiae

2.1 Library design and construction.

Promoters for this study were chosen to cover a wide variety of cellular functions and processes and to span various cellular compartments (Supp. material 1.1). Promoter sequences were defined as the genomic region located between the translation start site (TrSS) and the end of the upstream neighboring gene. Sequences larger than 1kbp were truncated to 1kbp to facilitate cloning. All strains were constructed as previously described, based on genomic integration of promoter sequences into a common master strain, upstream of a YFP reporter protein (Zeevi *et al*, 2011). A second mCherry reporter, driven by the constitutive TEF2 promoter was integrated to the same genomic location to control for the reliability of the strain construction process and experimental variability. Briefly, we used a master strain, based on Y8205, containing a construct of ADH1 terminator - mCherry - TEF2 promoter - Venus - ADH1 terminator - Nat1 on chromosome 15. Desired promoters were amplified by PCR from genomic DNA of BY4741 yeast strain, linked to a URA3 selection marker and integrated into the genome by homologous recombination. Final strains were validated by sequencing, growth curves, and mCherry expression levels, and abnormal strains were removed. In total, 867 strains were designed, and 859 were successfully constructed and measured (for a full list of promoters, primers, and sequences, see Table S1).

2.2 Promoter activity measurements.

Cells were inoculated from frozen stocks into synthetic complete dextrose (SCD) (150µl, 96 well plate) and grown at 30°C for 48 hours, reaching complete saturation. Cells were then diluted 1:36 in fresh medium to a total volume of 180µl and were grown at 30°C for at least 16 hours in 96-well plates while being measured. Measurements were carried out every 20 minutes using a robotic system (Tecan Freedom EVO) with a plate reader (Tecan Infinite F500). Each measurement included optical density (OD^{600}), YFP fluorescence, and RFP fluorescence. Measurements of each plate at every growth conditions were repeated 1-6 times.

2.3 Growth conditions.

The growth media in which all strains were measured are outlined in **Table S2**.

2.4 Computing promoter activity levels.

Basic analysis of measured OD, YFP, and RFP was done as previously described and included removal of strains with abnormal growth curves and RFP expression,

subtraction of background levels of OD and auto-fluorescence, and smoothing of outlier measurements for each strain (Zeevi *et al*, 2011). In order to calculate a promoter activity level that is comparable between conditions, for each measured plate we defined the time window of maximal growth. We identified the 20-minute interval of maximal growth ($\boldsymbol{\mu} = \mathbf{max}(\boldsymbol{dlog_2OD/dt})$) and extracted its doubling time ($\boldsymbol{\tau} = \mathbf{1}/\boldsymbol{\mu}$). We then searched for the window of two doubling times that includes the maximal point and has the lowest variance in $d\boldsymbol{OD}/\boldsymbol{dt}$, and calculated the average doubling time within this window. This stage was iterated until convergence, thus defining the time interval of maximal growth and the average doubling time within this time interval. For all conditions, convergence was achieved within a maximum of three iterations. For this time interval, we then computed the average YFP promoter activity per OD per second, as follows:

We regard a population of cells as a growing pool of biomass producing a fluorescent protein. We define:

- $OD(t)$ - is the population biomass at time t.
- $YFP(t)$ - is the total fluorescence in the population at time t.
- $pa(t)$- the promoter activity, is the fluorescence production rate per unit biomass at time t.

Since YFP is stable and long-lived, we can neglect its degradation and attribute its rate of accumulation to production alone: $YFP(t) = YFP(t_0) + \int pa(t')OD(t')dt'$. We now assume a phase of exponential steady state growth between t_1 and t_2 as described above. During this time $pa(t)$ is constant and thus $YFP(t) = YFP(t_0) + pa \int OD(t')dt'$. We get that $\boldsymbol{pa} = (\boldsymbol{YFP(t_2)} - \boldsymbol{YFP(t_1)})/\int_{\boldsymbol{t_1}}^{\boldsymbol{t_2}} \boldsymbol{OD(t')dt'}$ (Fig 1). To obtain promoter activities per OD per doubling time, values were multiplied by the average doubling time.

To compare promoter activity levels from multiple experiments, we had to account for technical variation between experiments. To this end, we performed a calibration experiment in which we measured the same strain (RPL3) in 12 replicates in all growth conditions. We repeated this experiment twice with freshly prepared media and randomized locations of conditions within the measurement plates. Average promoter activity levels for RPL3 for both experiments deviated by less than 5%. Each measurement plate in the library included four technical replicates of RPL3. We then scaled the promoter activity levels for all strains in each plate such that the median promoter activity of RPL3 for the plate was equal to that calculated in the calibration

experiment. For each strain in every condition, we took the final promoter activity levels to be the average of the strain across all measurement plates. If this average was below the detection level, we set the promoter activity to the detection level.

2.5 Estimating the detection level of our experimental system.

To assess the detection level of our system, we examined the distribution of promoter activity levels under all examined conditions for a strain containing an RFP gene but no YFP gene. For each condition, more than 30 biological replicates of the strain were measured and fitted to a normal distribution (**Fig. S1**), and the 95th percentile of the distribution was taken to be the detection level. All analyses were restricted to strains which are above detection level in at least one condition.

2.6 Experimental Variability.

To assess the experimental error of our system, we examined differences between biological replicates of promoter strains whose activities were above the detection threshold. The relative error was estimated by the coefficient of variation (CV) of replicate measurements. We then grouped the replicate measurements (42% of the measurements) into 20 equally-spaced bins (in logarithmic scale) according to their mean promoter activity. For each such group of similarly active promoters, we computed their mean CV and smoothed this mean by averaging the values of four neighboring bins. We then used these mean CV values for every bin to estimate the CV of any promoter activity level, by linear interpolation. The CV values ranged from 0.36 for very low promoter activity levels to 0.05 for high promoter activity levels (**Fig. S2**).

2.7 Global scaling factors and error model.

For all conditions the global scaling factor represents the best robust fit to the data and was identified by two separate methods: A) For each pairwise comparison, we performed a robust linear fit (linear fit ignoring outliers) using Matlab's *robustfit* function. B) Promoters were clustered as described below (section 2.10). Global scaling factors were then computed by finding the best linear fit to promoters of cluster 1. Both methods yielded similar scaling factors, deviating by less than 10%. Reported values in figures 2,S6 and table S4 are from the second method.

For each pairwise comparison we determined the variance explained by the global scale line. The promoter activity values, $\mathrm{v(p)}$, of each promoter p was projected to the global scale line. Denoting the difference between the vector and its projection by $d(p)$, the variance explained by the clustering was calculated as

$1 - \mathrm{variance}(\mathrm{d}(\mathrm{p})) / \mathrm{variance}(\mathrm{v}(\mathrm{p}))$. To obtain a p-value for the explained variance, for each comparison between conditions X and Y, we randomized the promoter activities of condition Y and quantified the variance observed by the original global scaling line, as described above. This was repeated 1000 times for each pairwise comparison, and p-values were computed under assumption of normal distribution.

For each pairwise comparison we then determined which promoters behave according to the global trend between these two conditions using two separate methods: A) We analyzed all data points above detection and estimated their probability to behave according to the global trend. For each promoter, let (x, y) and (sx, sy) denote its activity level and standard deviation in conditions x and y, respectively. Denoting the scaling factor between the respective conditions by a, then promoters were defined as part of the global trend if $|\mathrm{ax} - \mathrm{y}| < 3\mathrm{sy}$ or $|\mathrm{x} - \mathrm{y}/\mathrm{a}| < 3\mathrm{sx}$. B) We restricted each pairwise comparison to promoters with an activity greater than 0.1 in both conditions. For such values we found the average CV to remain constant at 0.05 (**Fig. S2**). For each pairwise comparison we defined a promoter as part of the global trend if its value deviated by no more than 30% from its expected value according to the global scaling factor.

These two methods complement each other as the first is relative yet it enables the analysis of the entire dataset, taking into account our different level of confidence in low and high values. The second is restricted to only parts of the data, yet it enables to determine absolute values. Both yielded similar results with ~60-90% of promoters (depending on the pair of conditions compared) changing between conditions according to the global scaling factor.

2.8 Quantitative PCR analysis.

18 representative strains belonging to cluster 1 (RPB10, TEF1, DPM1, SEC61, SHP1, CDC10, RPS3, GLY1, RPL3, RPL33A, RPL8B, RPS7A, RPS11B, RPL4B, RPL28) or cluster 6 (GAL1, GAL2, GAL7) were inoculated from frozen stocks into synthetic complete dextrose (SCD) (150µl, 96 well plate) and grown at 30°C for 48 hours, reaching complete saturation. Cells were then diluted 1:36 in fresh medium to a total volume of 180µl and were grown at 30°C 96-well plates while being measured for OD, RFP, and YFP. At mid exponential phase 8 wells (~1ml) from each strain were united, centrifuged and pelleted cells were frozen at -80°C. RNA was extracted using EPICENTER Yeast MasterPURE RNA extraction kit, and cDNA was created using random hexamers (sigma). cDNA samples were diluted 1:10 and quantitative PCR was

performed by RT-PCR (StepOnePlus, Applied Biosystems) using a ready-mix kit (KAPA, KK4605). A standard curve was prepared from serial dilutions of a mixture of all strains. For each strain, measurements were performed in two sets of triplicates, measuring both YFP (primers: CCAGAAGGTTATGTTCAA , CGATTCTATTAACTAAGGTATC) and RFP mRNA (primers: TGTGGGAGGTGATGTCCAACTTGA , AGATCAAGCAGAGGCTGA-AGCTGA). Reported values are of mean YFP/RFP from 9 replicates derived from 3 independent experiments.

2.9 Functional annotation and enrichment analysis.

Sets of genes were assigned process, function, and cellular components according to the annotations from the Gene Ontology (GO) (Ashburner *et al*, 2000). The significant representation of GO terms in the set was evaluated by Gorilla GO Term Finder (Eden *et al*, 2009) with a p-value threshold of 10^{-3}. For TF analysis we examined the distribution of known TF promoters (Badis *et al*, 2008; Zhu *et al*, 2009) across the different clusters. For enrichment analysis, promoters were classified as previously described according to their properties as: OPN/DPN (Tirosh & Barkai, 2008), SAGA-dominated/TFIID-dominated (Huisinga & Pugh, 2004), divergent/unique (this study, based on (Saccharomyces Genome Database). Enrichment p-values were computed according to the HG distribution and corrected for multiple hypothesis testing using false discovery rate correction (Benjamini & Hochberg, 1995).

2.10 Clustering promoter activities.

To partition the promoters into clusters that preserve proportionality, we used K-means clustering with the cosine metric (defined by $(\mathrm{x},\mathrm{y}) = 1 - \cos(\sphericalangle \mathrm{x}0\mathrm{y}) = 1 - \frac{\mathrm{x}\cdot\mathrm{y}}{||\mathrm{x}||\cdot||\mathrm{y}||}$, where x and y are vectors of promoter activity levels in a given condition and O is the origin). The cosine metric ensures that two promoters whose activity levels across all conditions are equal up to some scaling factor, will have distance zero, and will thus reside in the same cluster. The clustering was repeated 100 times with different random starting points and the clustering that minimized the sum of distances from the centers was chosen. The number of clusters, K, was determined as the largest K for which the distance between any two centers is at least 0.05 (**Fig. S8**), thereby ensuring a minimal separation between any two clusters. Promoters that had very low activity levels (mean activity across conditions < 1.5 X mean detection level across all conditions) were excluded from the clustering (127 promoters). Promoters that had missing values in part of the conditions (90 promoters) were clustered separately after the initial clustering.

Each such promoter was assigned to the closest center using the cosine metric reduced to known coordinates.

For generation of figure S10, this analysis was repeated excluding all ribosomal promoters.

2.11 Variance explained by clustering.

For each promoter p, its vector of promoter activity levels across conditions, $v(p)$, was projected to the center of the corresponding cluster. Denoting the difference between the vector and its projection by $d(p)$, the variance explained by the clustering was calculated as $1 - \mathrm{variance(d(p))} / \mathrm{variance(v(p))}$.

2.12 Predicting promoter activity levels.

We used the following scheme to predict promoter activity levels under growth condition Y from measurements of several other conditions $\mathrm{x_1, \ldots, x_m}$. First, the number of clusters k for all promoters under the measured m conditions was determined using above criterion. Then, the promoters were clustered by the k-means algorithm using the cosine metric. Denote the centers of the clusters by $c_1, \ldots, c_k$. Since the cosine metric is invariant to scaling, we can assume that the norm of each center equals one. Any promoter that was measured only under part of the m conditions was assigned to the closest center, using the cosine metric reduced to known coordinates. The missing values of such promoters were estimated by $x(i) = \frac{c_t(i)}{\sqrt{\sum_{x(j) is\ known} c_t(j)^2}} \cdot \sqrt{\sum_{x(j) is\ known} x(j)^2}$, where x is the vector of size m of promoter activities for the specific gene, c_t is the center of the cluster to which the gene belongs, and i is an index of missing value in x. After obtaining the full matrix of promoter activities for all genes under $\mathrm{x_1, \ldots, x_m}$, without missing values, the centers of the clusters $c_1, \ldots, c_k$ were recalculated.

A small number of representative promoters were chosen as the training set, and their promoter activity levels under the new condition Y were used for the prediction task. For each cluster t, an extended center $\widehat{c_t}$ of size $m + 1$was calculated from the representative promoters that belong to cluster t (if no representative genes belonged to cluster t, then the extended center of the largest cluster was used) and normalized to have a norm of one. Then, $\widehat{c_t}$ was used for prediction of promoter activity of all other genes from cluster t under the new condition Y. The activity level of a promoter was predicted to be $\frac{\widehat{c_t}(m+1)}{\sqrt{\sum_{j=1}^{m} \widehat{c_t}(j)^2}} \cdot \sqrt{\sum_{j=1}^{m} x(j)^2}$, where x is the vector of size m of activity levels

for that promoter. The representative promoters were chosen as follows: an equal number of promoters were chosen from each cluster, which are closest (by the cosine metric) to the centers $c_1, \ldots, c_k$ of the relevant clusters. If the number of requested representatives is not divisible by k, then the remainder promoters are chosen from the largest clusters.

3. Supplementary methods- E. coli

3.1 Growth conditions.

All media for bacterial growth were based on a defined M9 minimal medium (42mM Na2HPO4, 22mM KH2PO4, 8.5mM NaCl, 18.5mM NH4Cl, 2mM MgSO4, 0.1mM CaCl, 16µM Thiamine)+50µg/ml kanamycin. Specific growth conditions and the respective growth rates in each condition are listed in **Table S8**.

3.2 Robotic assay for genome-wide promoter activity data.

The library of reporter strains, each bearing a low-copy plasmid with one of *E .coli* promoters controlling fast-folding GFP (**Fig. S14A**, (Cormack *et al*, 1996)) was previously described (Zaslaver *et al*, 2006). This library includes 1820 reporter strains which represent ~75% of E. coli promoters. Reporter strains were inoculated from frozen stocks into in high-brim 96-well plates containing M9 minimal medium supplemented with 11mM glucose, 0.05% casamino acids and 50µg/ml kanamycin. The 96-well plates were covered with breathable sealing films (Excel Scientific Inc.) and grown overnight in a shaker at 37°C. All steps from this point were carried out using a programmable robotic system (Freedom Evo, Tecan Inc.). Overnight cultures were first diluted 1:33 times into M9 medium followed by a second 1:15 dilution into 6 flat bottom microwell plate (nunc) containing one of the growth media (**Table S8**) in a final culture volume of 150µl. Wells were then covered with 100µl of mineral oil (Sigma) to prevent evaporation and transferred into an automated incubator. Bacteria were grown in the incubator with shaking (6 Hz) at 37°c for about 20 hr. Every 8 min the plate was transferred by the robotic arm into a multiwall fluorometer (Infinite F200, Tecan) that reads the OD (600 nm) and GFP (excitation 480 (20), emission 515(10)). After 5 hours of incubation NaCl or casamino were added to the appropriate plates by automated pipetting below the oil layer. Each plate contained several control strains: Promoterless strain used for the subtraction of auto-fluorescence background (Zaslaver *et al*, 2006); Sigma70 synthetic promoter bearing the consensus sigma70 binding site (Kaplan *et al*, 2008); GadB promoter which was used a representative of a sigmaS regulated promoter (Keseler *et al*, 2011).

3.3 Computing promoter activity levels, detection level, experimental variability and error model.

Promoter activity was calculated by the rate of GFP production per OD unit, as described above for yeast (**supp. methods 2.4**) for the 3-hour window around mid-

exponential growth (**Fig. S14**). For conditions in which a compound was added to the media, promoter activity was calculated for the window of time after its addition. Background fluorescence was measured using a promoter-less control strain in each plate. Promoter activities lower than 3 STDs above the mean background promoter activity were set to zero. We find that about 300-500 promoters are active above background in each condition, and 100 promoters are active in all conditions. In total, 969 promoters were active above background in at least one condition. Experimental variability was assessed as described above, using three replicate measurements in M9 glucose (**Fig. S15**) and error model was calculated as for *S. cerevisiae*.

Identification of representative promoters for predictions was done iteratively. At each iteration, we calculated the best linear sum of the representative promoter, which predicted the experimental data, and added an additional representative promoter, which contribute the most to predict the experimental data.

4. Supplementary figures

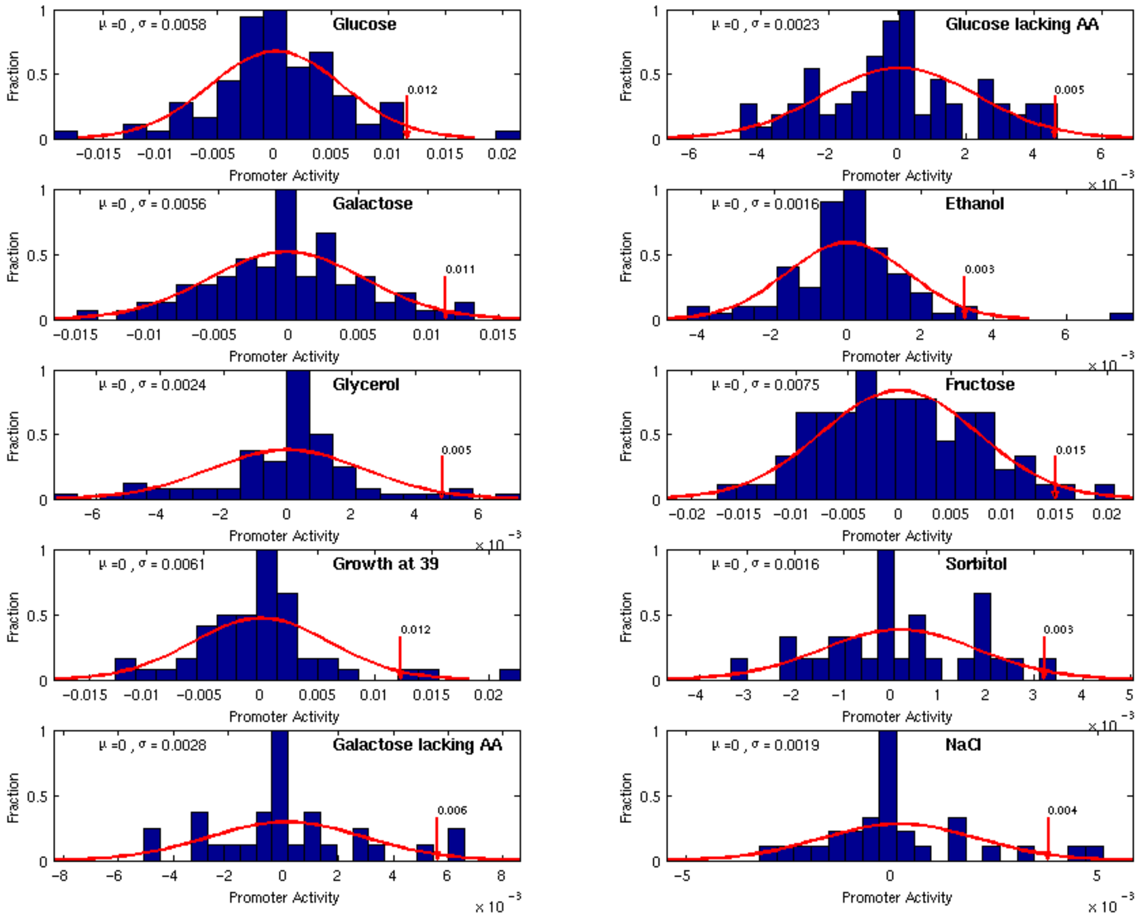

Supplementary figure 1. Estimating the background level of promoter activity. Shown is the distribution of YFP promoter activities across all examined growth conditions, for a strain that contains a mCherry reporter gene but no YFP reporter gene. For each condition (upper right text), shown is a histogram (blue bars) of the fraction of over 30 biological replicate measurements as well as a fit of the histogram to a normal distribution (red line). The 95th percentile of the distribution (red arrow) was set as the level above which YFP-containing strains are considered to be above background.

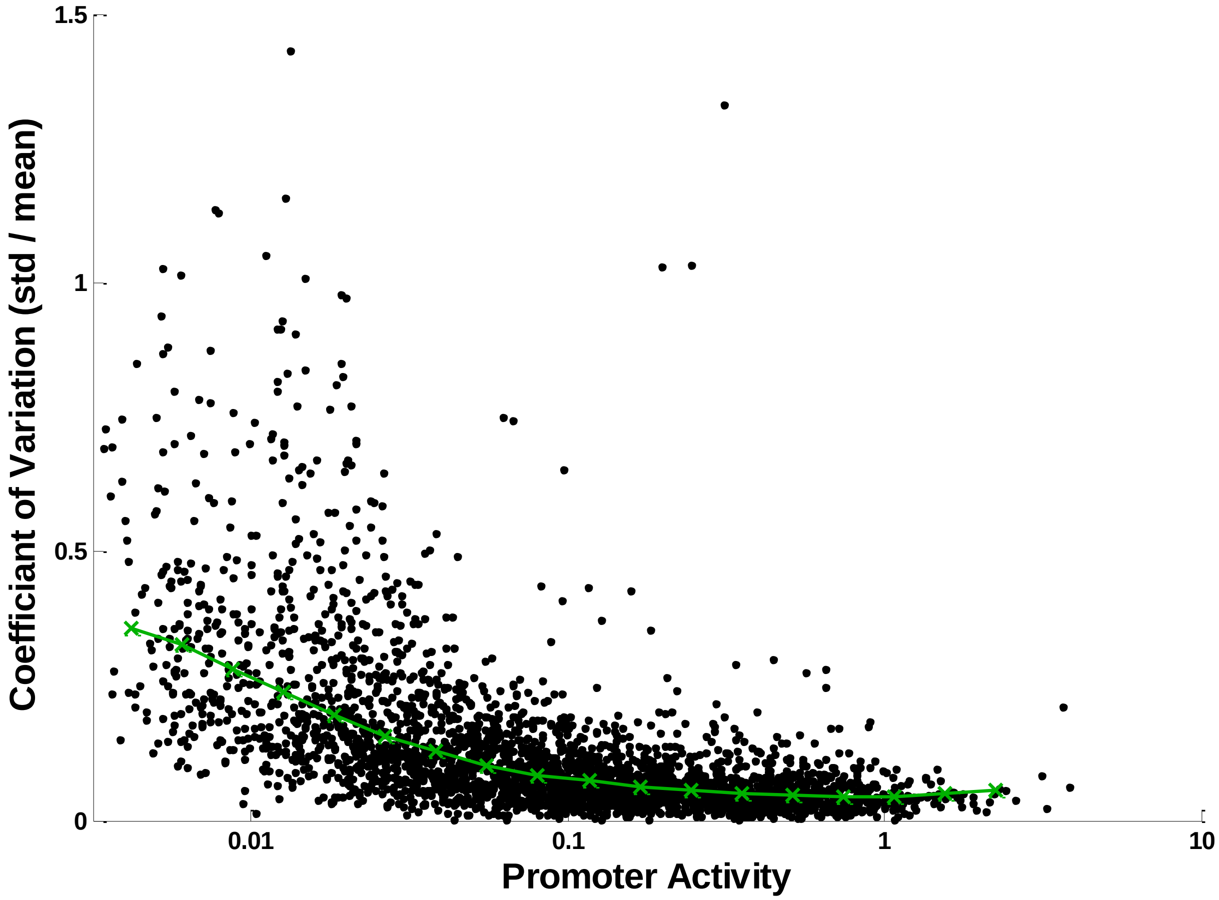

Supplementary figure 2. Estimating the experimental variability of our system. For each promoter which was measured in replicates (42% of the promoters), shown is the coefficient of variation (CV, standard-deviation divided by the mean, y-axis) of its activity against its mean activity, where the activity level of each promoter was derived from 2-6 replicates. The measurements were grouped by their promoter activity into 20 equally-spaced bins (in logarithmic scale, green X marks). Also shown (green curve) is a linear interpolation of the CV of each promoter activity using the CVs of four neighboring bins. We used this linear interpolation as an estimate for the relative error of promoters that were not measured in replicates.

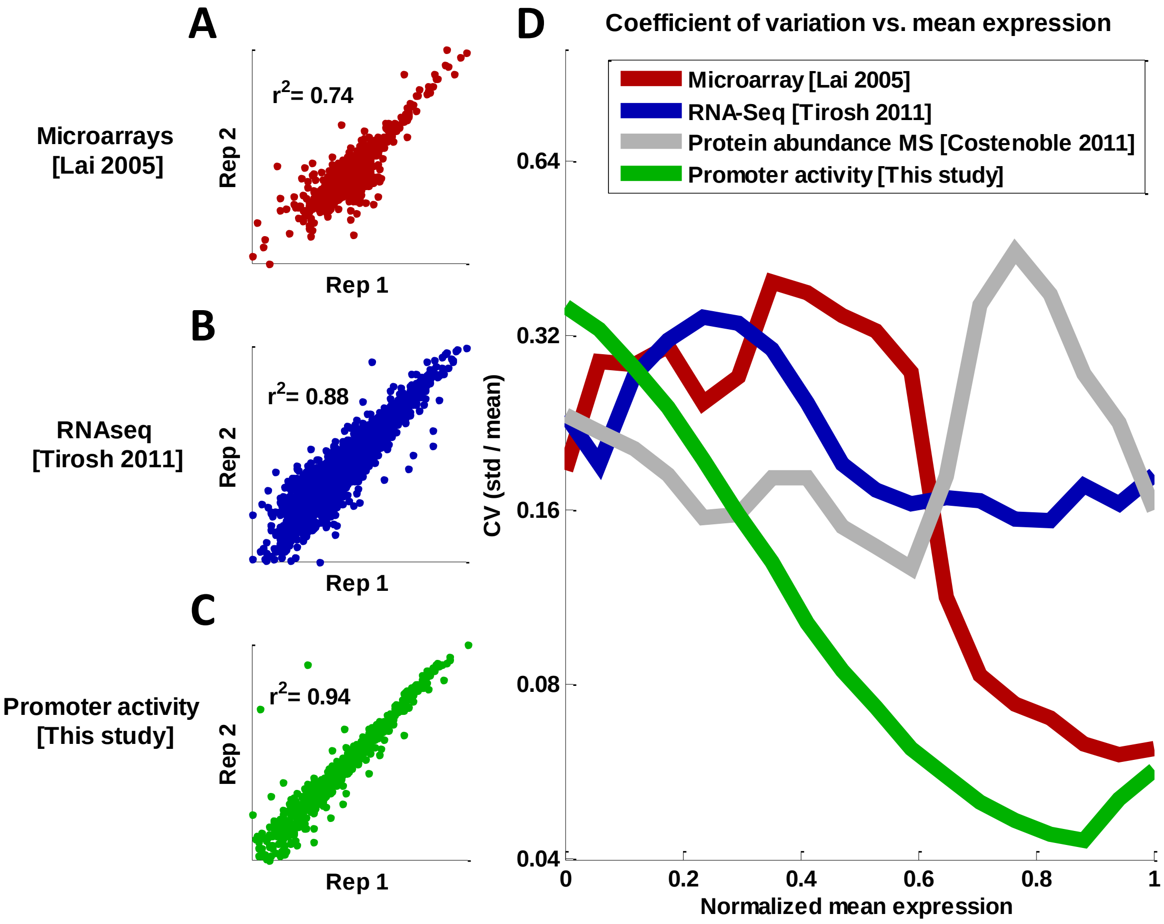

Supplementary figure 3. Comparing the experimental variability of our system to existing techniques . Shown is a comparison of two biological replicates, measured by **(A)** microarrays (Lai *et al*, 2005), **(B)** RNA sequencing (Tirosh *et al*, 2011) and **(C)** fluorescence reporters (this study). (**D**) For the datasets from A-C and an additional mass spectrometry dataset (Costenoble *et al*, 2011) shown is the coefficient of variation (CV, standard-deviation divided by the mean, y-axis) against mean expression level (x-axis). For each dataset, the measurements were grouped by their mean levels into 20 equally-spaced bins (in logarithmic scale) and mean CV per bin was interpolated using the CVs of four neighboring bins. For all datasets mean expression levels were normalized to be from 0 to 1.

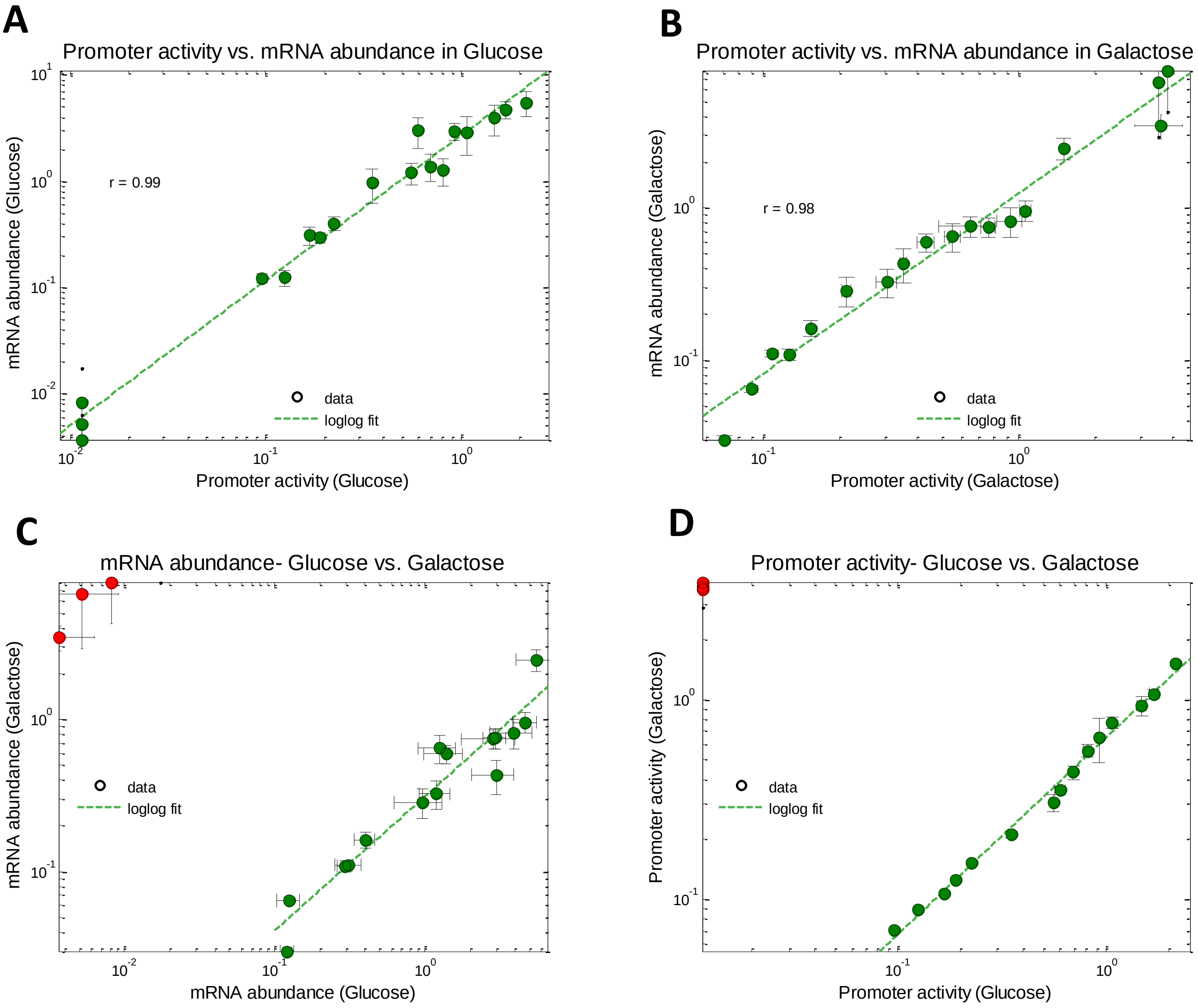

Supplementary figure 4. Protein levels of YFP are a good proxy for mRNA levels. **(A and B)** For 18 different promoter strains from our library (see supp. material for identities of promoters) grown in either glucose (A) or galactose (B), shown are mRNA levels measured by quantitative PCR (y-axis) and the corresponding promoter activity levels calculated from fluorescence as described (x-axis). For both conditions we found high correlation between mRNA and promoter activity (top left). **(C and D)** Comparison of mRNA levels (C) or promoter activities (D) in both conditions shows proportional scaling for genes belonging to cluster 1 (green dots), whereas condition-specific promoters (cluster 6, red dots) are differentially activated between the two conditions.

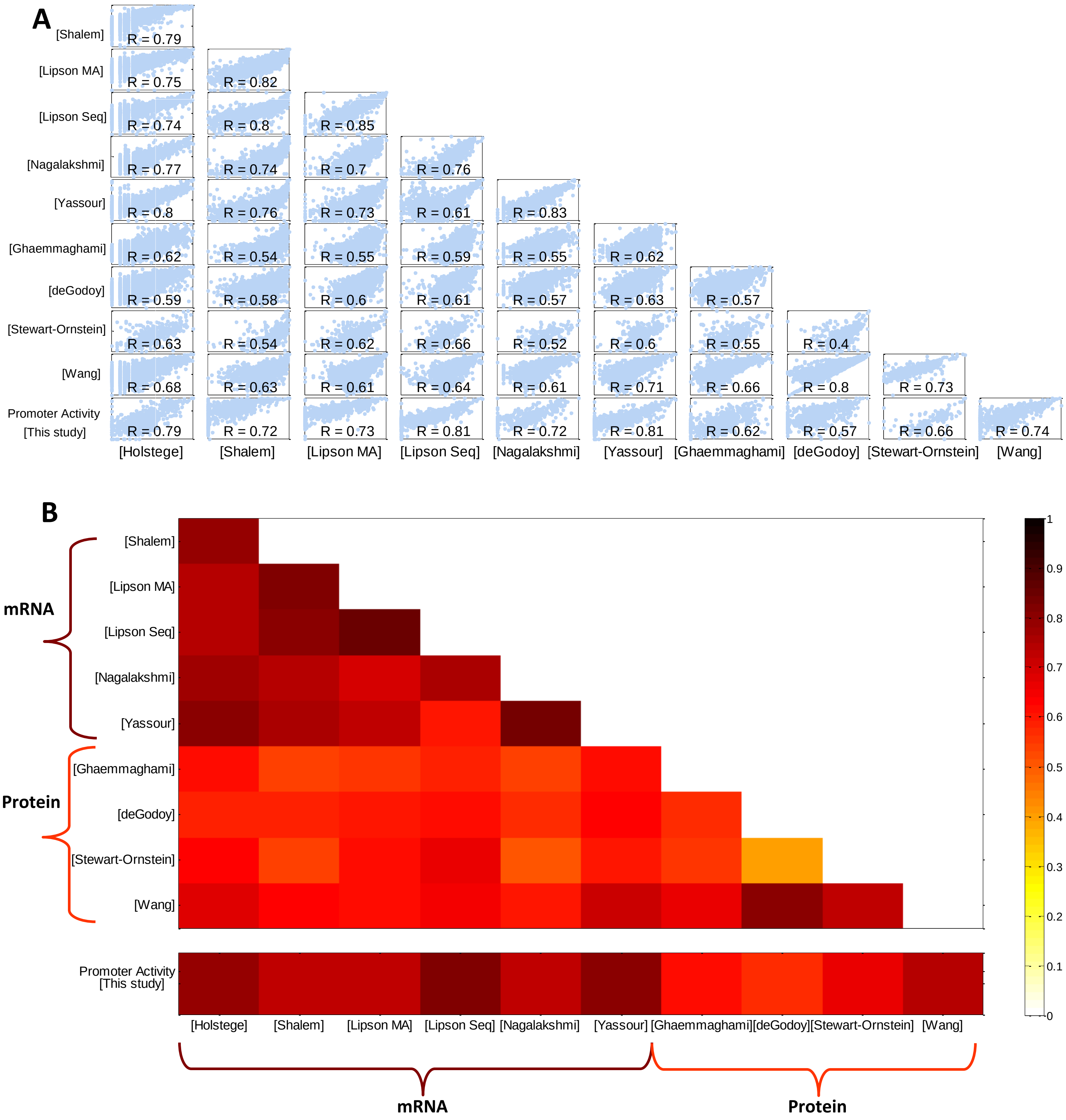
A
[Shalem]
[Lipson MA]
[Lipson Seq]
[Nagalakshmi]
[Yassour]
[Ghaemmaghami]
[deGodoy]
[Stewart-Ornstein]
[Wang]
Promoter Activity
[This study]
R = 0.79
R = 0.75
R = 0.82
R = 0.74
R = 0.8
R = 0.85
R = 0.77
R = 0.74
R = 0.7
R = 0.76
R = 0.8
R = 0.76
R = 0.73
R = 0.61
R = 0.83
R = 0.62
R = 0.54
R = 0.55
R = 0.59
R = 0.55
R = 0.62
R = 0.59
R = 0.58
R = 0.6
R = 0.61
R = 0.57
R = 0.63
R = 0.57
R = 0.63
R = 0.54
R = 0.62
R = 0.66
R = 0.52
R = 0.6
R = 0.55
R = 0.4
R = 0.68
R = 0.63
R = 0.61
R = 0.64
R = 0.61
R = 0.71
R = 0.66
R = 0.8
R = 0.73
R = 0.79
R = 0.72
R = 0.73
R = 0.81
R = 0.72
R = 0.81
R = 0.62
R = 0.57
R = 0.66
R = 0.74
[Holstege]
[Shalem]
[Lipson MA]
[Lipson Seq]
[Nagalakshmi]
[Yassour]
[Ghaemmaghami]
[deGodoy]
[Stewart-Ornstein]
[Wang]
B
mRNA
Protein
[Shalem]
[Lipson MA]
[Lipson Seq]
[Nagalakshmi]
[Yassour]
[Ghaemmaghami]
[deGodoy]
[Stewart-Ornstein]
[Wang]
Promoter Activity
[This study]
[Holstege]
[Shalem]
[Lipson MA]
[Lipson Seq]
[Nagalakshmi]
[Yassour]
[Ghaemmaghami]
[deGodoy]
[Stewart-Ornstein]
[Wang]
mRNA
Protein
1
0.9
0.8
0.7
0.6
0.5
0.4
0.3
0.2
0.1
0

Supplementary figure 5. Promoter activities are correlated with mRNA levels and protein abundance. Shown are scatter plots **(A)** or a heat-map **(B)**, displaying the correlations between promoter activities under glucose (this study, x-axis) and published mRNA or protein abundance measurements in mid-log glucose conditions using a variety of experimental technologies (y-axis), consisting of microarrays (Holstege *et al*, 1998; Shalem *et al*, 2008; Lipson *et al*, 2009), RNA sequencing (Lipson *et al*, 2009; Nagalakshmi *et al*, 2008; Yassour *et al*, 2009), immune-tagged proteins (Ghaemmaghami *et al*, 2003), fluorescently-tagged proteins (Stewart-Ornstein *et al*, 2012), mass spectrometry (de Godoy *et al*, 2008) and a curated dataset of protein abundances integrated from 5 different datasets (Wang *et al*, 2012). The correlation coefficients between promoter activity and these datasets are similar to the correlation between these datasets. As expected, the mRNA datasets better correlate between themselves than they do with the protein datasets. Promoter activity correlates better with mRNA levels than with protein levels.

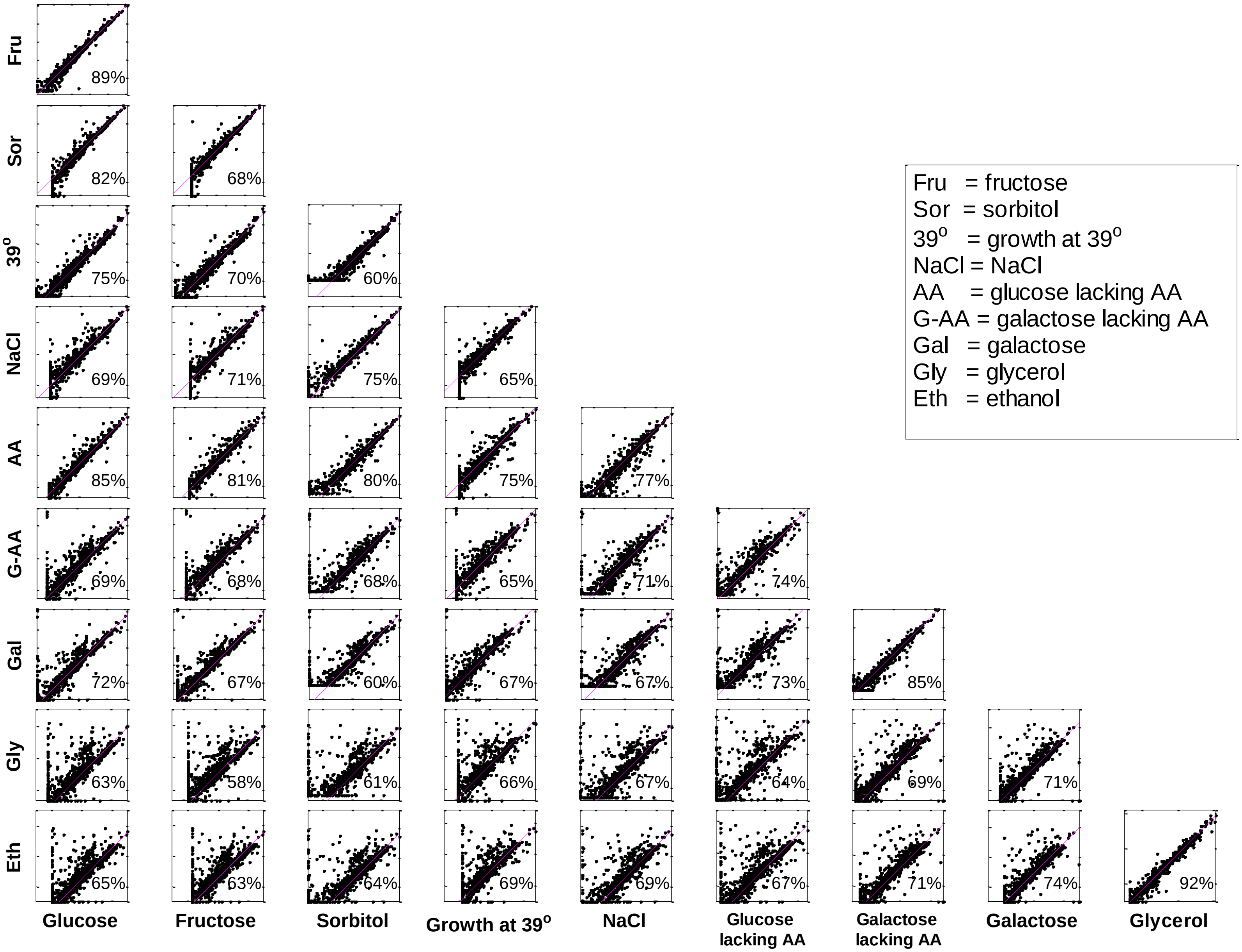

Supplementary figure 6. Most promoters preserve their relative activity levels between every pair of growth conditions. Shown is a comparison of promoter activities between every pair of tested conditions. The slope of the robust linear fit (magenta line) represents the scaling factor between the two conditions. The percent of promoters that deviate less than 3 standard deviations from the global trend is indicated for each pair of growth conditions.

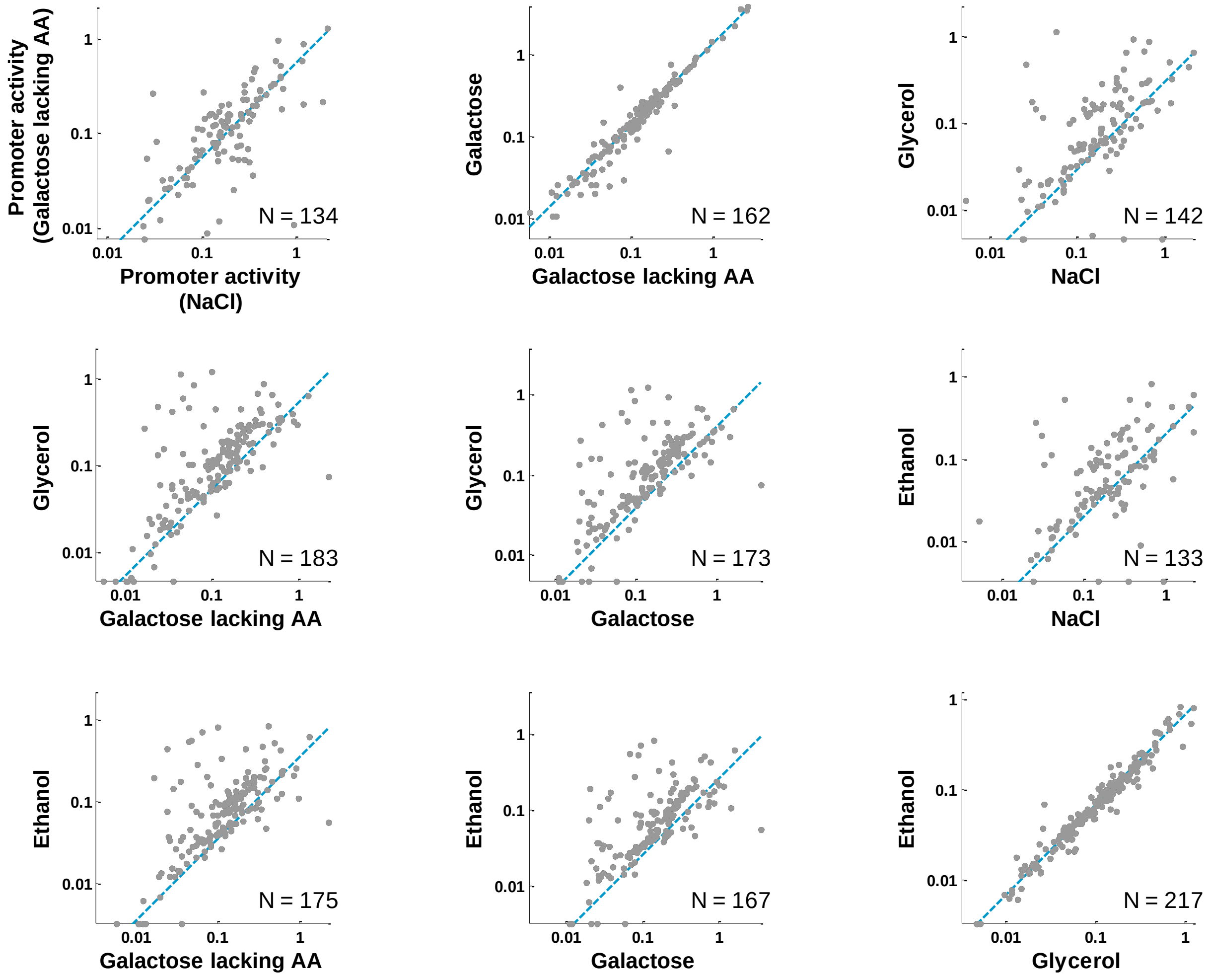

Supplementary figure 7. Condition-specific promoters preserve their relative activity levels between conditions in which they are activated. For every pair of conditions with an overlap of at least 50% in their set of condition-specific promoters, shown is a comparison of the activities of these N overlapping promoters. For each condition, its condition-specific promoters are defined as those whose activities differ by more than 3 standard deviations from the global trend of its comparison to glucose. In all pairwise comparisons shown, promoters display proportional activities, as indicated by their alignment to straight lines. In some cases, the scaling of the condition-specific promoters coincides with the global trend (cyan dashed line) of the two conditions being compared, while in others the scaling factor differs.

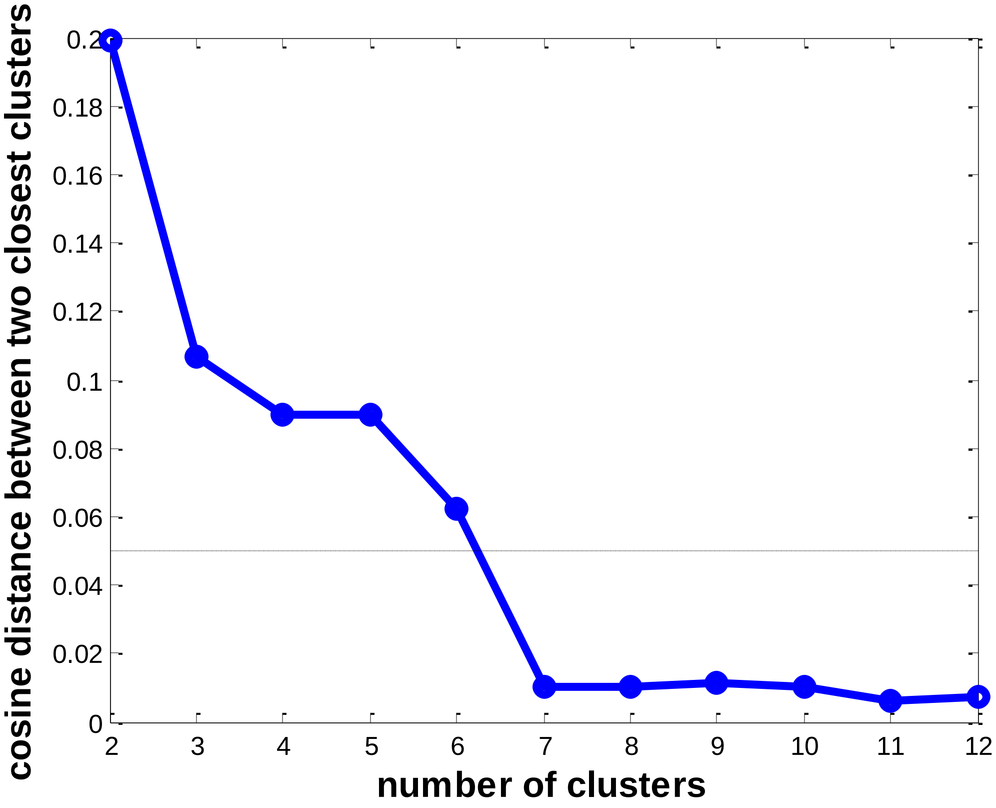

Supplementary figure 8. Choosing the number of clusters for K-means clustering. To select the number of clusters, we clustered the activities of all promoters under all measured conditions into 2 to 12 clusters using K-means with the cosine distance function (Methods). Shown is the cosine distance (1 – cosine of angle) between the two closest centers (y-axis), as a function of the number of clusters (x-axis). Since clustering the data into 7 clusters resulted in two clusters that are nearly indistinguishable (the cosine distance between their centers is ~ 0.01), we used 6 clusters in our subsequent analyses.

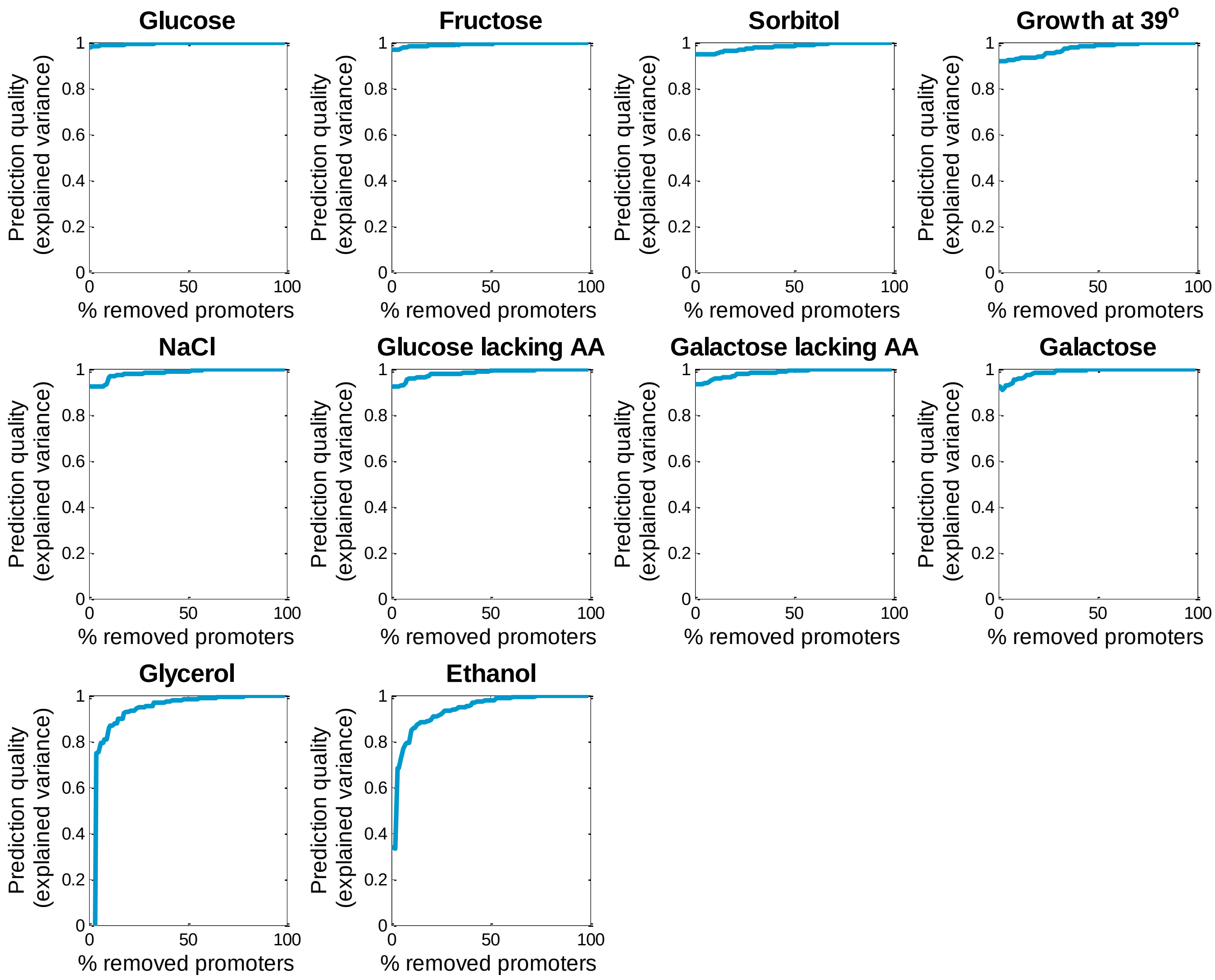

Supplementary figure 9. Promoter activities can be predicted with high accuracy using only a few representative promoters. For each growth condition, shown is the fraction of the data variance explained by the predicted promoter activities (y-axis) when predictions are generated using the promoter activities of all other growth conditions. This fraction is shown when removing the *k* percent of the promoters that are predicted worst (x-axis) for all values of *k*. Predictions were generated using a subset of 10 promoters, chosen based on their distance from the centers of the clusters (Methods). In all conditions, more than 85% of the variation in the data is explained upon removal of at most 10 promoters.

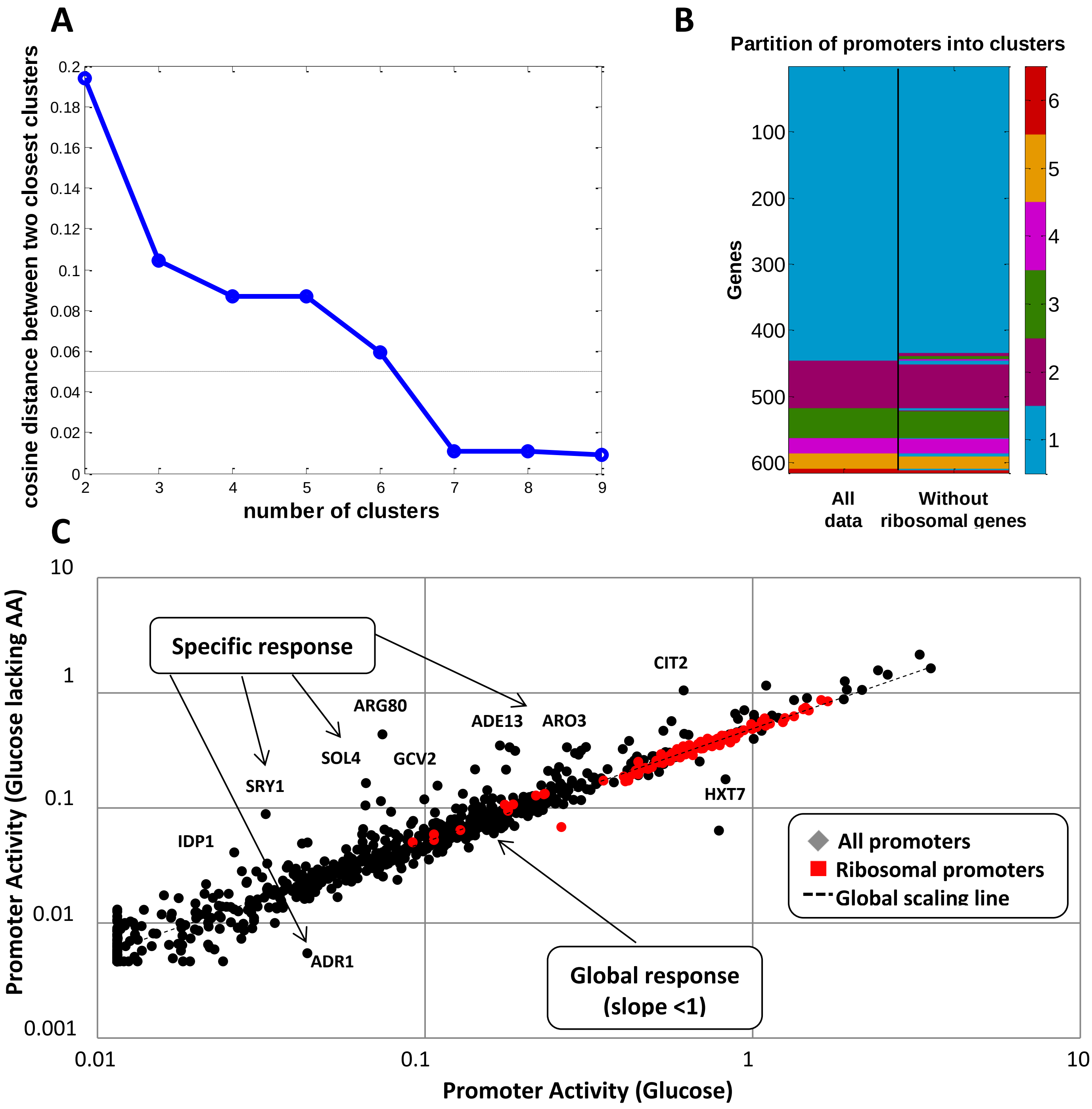
A
cosine distance between two closest clusters
number of clusters
B
Partition of promoters into clusters
Genes
All data
Without ribosomal genes
C
Specific response
ARG80
SOL4
GCV2
SRY1
IDP1
ADR1
ADE13
ARO3
CIT2
HXT7
All promoters
Ribosomal promoters
Global scaling line
Global response (slope <1)
Promoter Activity (Glucose lacking AA)
Promoter Activity (Glucose)

Supplementary figure 10. Clustering of promoter activities without ribosomal promoters. Clustering analysis was repeated, without the 110 promoters of the ribosomal proteins. (**A**) Number of clusters was chosen as in Figure S8, by enumerating over a range of possibilities and selecting the largest number that still guarantees well-separated clusters. (**B**) Clustering of promoters into 6 clusters using the cosine metric (Methods) was performed for all promoters (left) or excluding ribosomal promoters (right), resulting in highly similar results, whereby 95% of promoters are clustered the same using both methods. Colors represent the different clusters. (**C**) Shown is the promoter activity in glucose (x-axis) and glucose lacking amino acids (y-axis). Ribosomal promoters, excluded from the clustering analysis, are highlighted in red. Dashed line indicates the scale line obtained from clustering the data without ribosomal promoters. Notably, this scale line also accurately accounts for the excluded promoters.

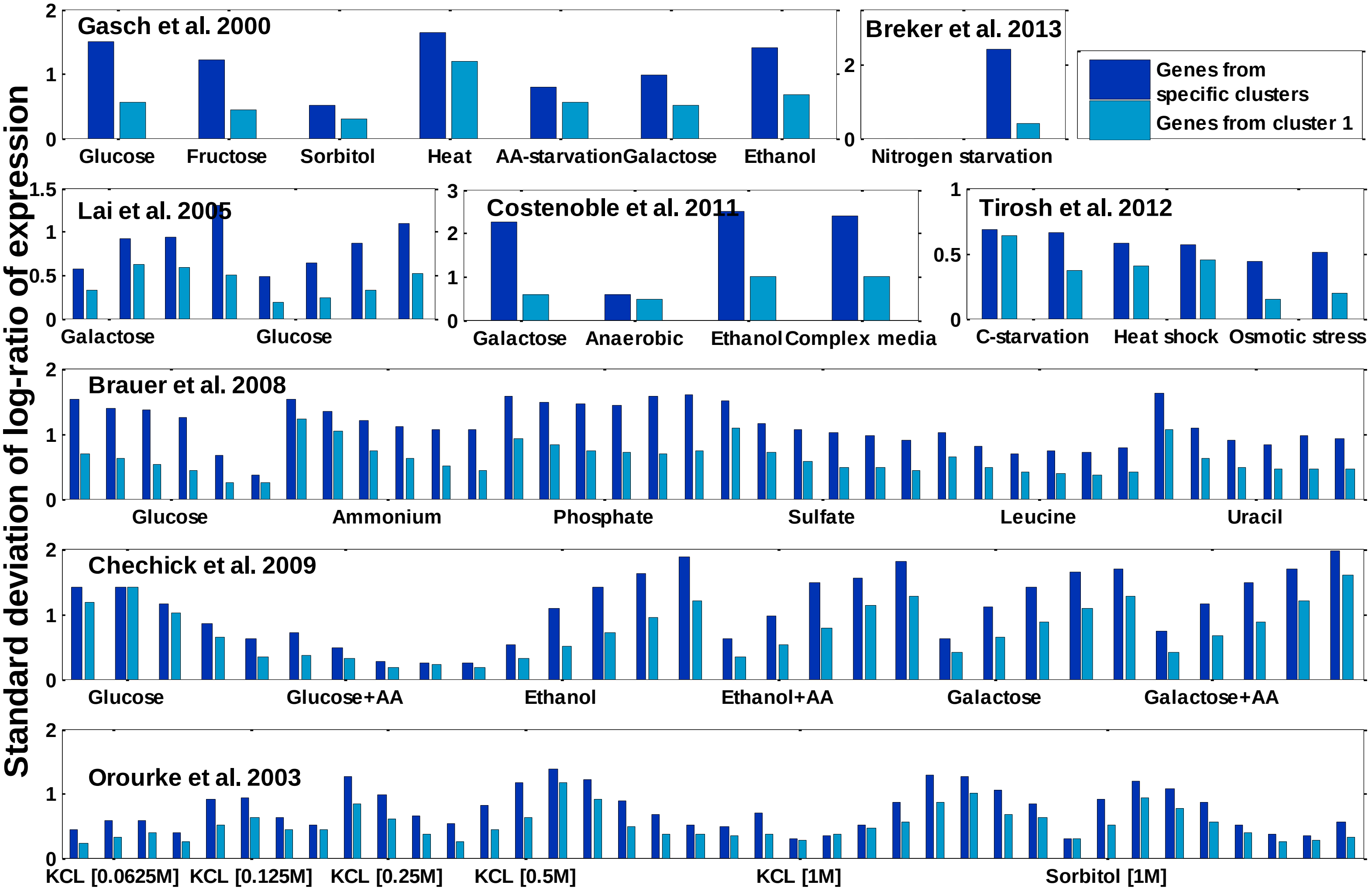
Standard deviation of log-ratio of expression
Gasch et al. 2000
Glucose
Fructose
Sorbitol
Heat
AA-starvation
Galactose
Ethanol
Breker et al. 2013
Nitrogen starvation
Genes from specific clusters
Genes from cluster 1
Lai et al. 2005
Galactose
Glucose
Costenoble et al. 2011
Galactose
Anaerobic
Ethanol
Complex media
Tirosh et al. 2012
C-starvation
Heat shock
Osmotic stress
Brauer et al. 2008
Glucose
Ammonium
Phosphate
Sulfate
Leucine
Uracil
Chechick et al. 2009
Glucose
Glucose+AA
Ethanol
Ethanol+AA
Galactose
Galactose+AA
Orourke et al. 2003
KCL [0.0625M]
KCL [0.125M]
KCL [0.25M]
KCL [0.5M]
KCL [1M]
Sorbitol [1M]

Supplementary figure 11. The intra-cluster variability is smaller than the inter-cluster variability in genome-wide mRNA and protein measurements. For over 100 genome-wide mRNA or protein measurements in yeast, derived from 7 different studies, shown is the standard deviation of reported log-ratio expression levels of two sets of genes: (1) genes whose promoter activities were clustered into the first global response cluster in our study (right column, cyan); and (2) genes whose promoter activities were clustered into any of the other five clusters (left column, blue). In all cases we found the intra-cluster variability to be lower than the inter-cluster variability, as expected if proportional scaling is largely preserved for the mRNA and protein levels. Conditions include: **(A)** Gasch et al., 2000, genome-wide expression mRNA measurements using microarrays of seven conditions that are similar to growth conditions that we tested. **(B)** Breker et al., 2013, genome-wide microscopy data for fluorescently-tagged fusion proteins in glucose and nitrogen starvation. **(C)** Lai et al., 2005, Time-course data for glucose and galactose using microarrays. **(D)** Costenoble et al., 2011, ~200 proteins measured in the indicated conditions by mass spectrometry. **(E)** Brauer et al., 2008, mRNA measurements using microarrays for 36 steady-state continuous cultures limited by one of six different nutrients. **(F)** Chechick et al., 2009, Time-course data for 6 different environmental conditions using microarrays. **(G)** Orourke et al., 2003, Time-course data for different concentrations of osmotic stress using microarrays.

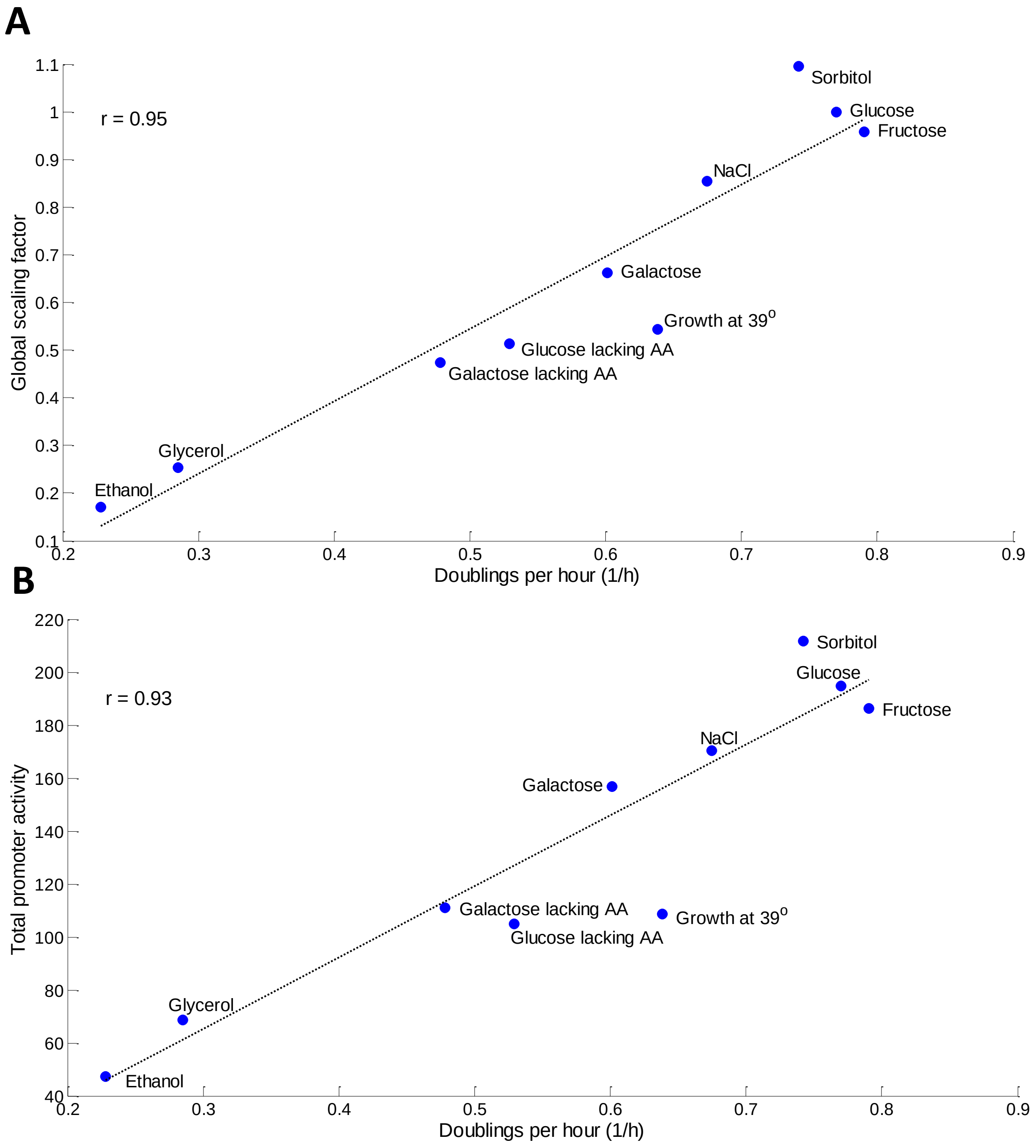

Supplementary figure 12. Global scaling factors and total promoter activity are correlated to growth rate. For each growth condition, shown is the growth rate (x-axis) and (**A**) global scaling factor or (**B**) total promoter activity (y-axis). The scaling factor for glucose was arbitrarily set to 1.

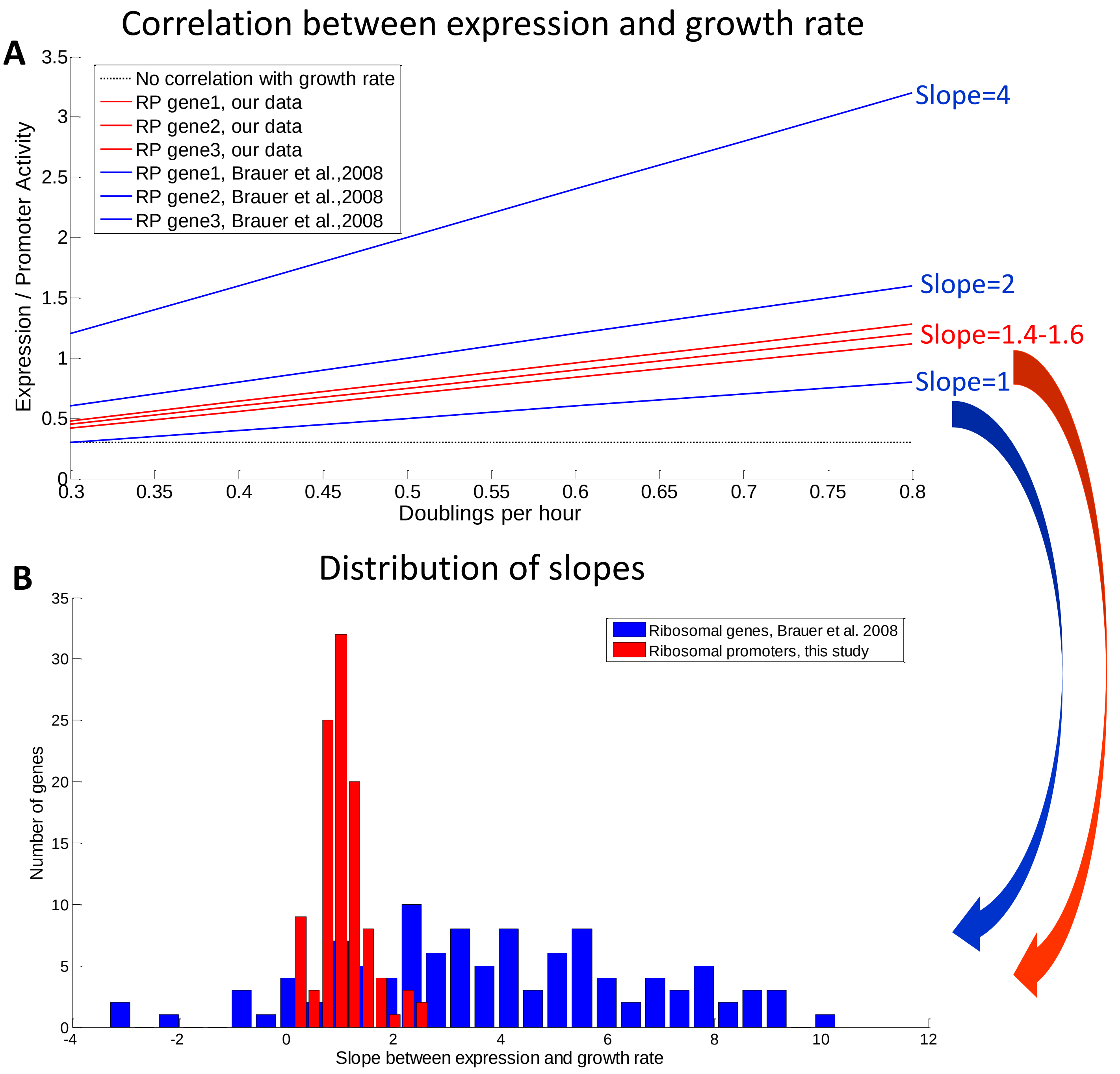

Correlation between expression and growth rate
A
No correlation with growth rate
RP gene1, our data
RP gene2, our data
RP gene3, our data
RP gene1, Brauer et al.,2008
RP gene2, Brauer et al.,2008
RP gene3, Brauer et al.,2008
Slope=4
Slope=2
Slope=1.4-1.6
Slope=1
Expression / Promoter Activity
Doublings per hour
B
Distribution of slopes
Ribosomal genes, Brauer et al. 2008
Ribosomal promoters, this study
Number of genes
Slope between expression and growth rate

Supplementary figure 13. Correlation between expression/promoter activity and growth rate

(**A**) For 110 ribosomal genes we found the slope between their expression (Brauer *et al*, 2008) or promoter activity and growth rate. Plotted are 3 illustrative examples from each dataset. Slopes for expression data (blue lines) were taken from (Brauer *et al*, 2008) and slopes for promoter activities (red lines) were obtained by fitting a linear function (using matlab's *polyfit*) for each gene between promoter activity values (Table S3) and growth rates (Table S4). Ribosomal proteins were chosen as their expression was shown to correlate with growth rate in many different studies (Regenberg *et al*, 2006; Castrillo *et al*, 2007; Brauer *et al*, 2008; Fazio & Jewett, 2008; Neidhardt, 1999; Bremer & Dennis, 1987; Zaslaver *et al*, 2009; Klumpp *et al*, 2009; Levy & Barkai, 2009; Scott *et al*, 2010; Pedersen *et al*, 1978). Shown are 3 representative examples from both datasets. (**B**) Shown is the distribution of slopes between expression (blue bars) or promoter activity (red bars) and growth rate for 110 ribosomal genes. We note that both we and (Brauer *et al*, 2008) observe a positive correlation of ribosomal gene expression with growth rate, as indicated by the positive slopes for most ribosomal genes using both methods. However, our data additionally suggests that the magnitude of this correlation (i.e., the slopes) is highly similar for all ribosomal genes.

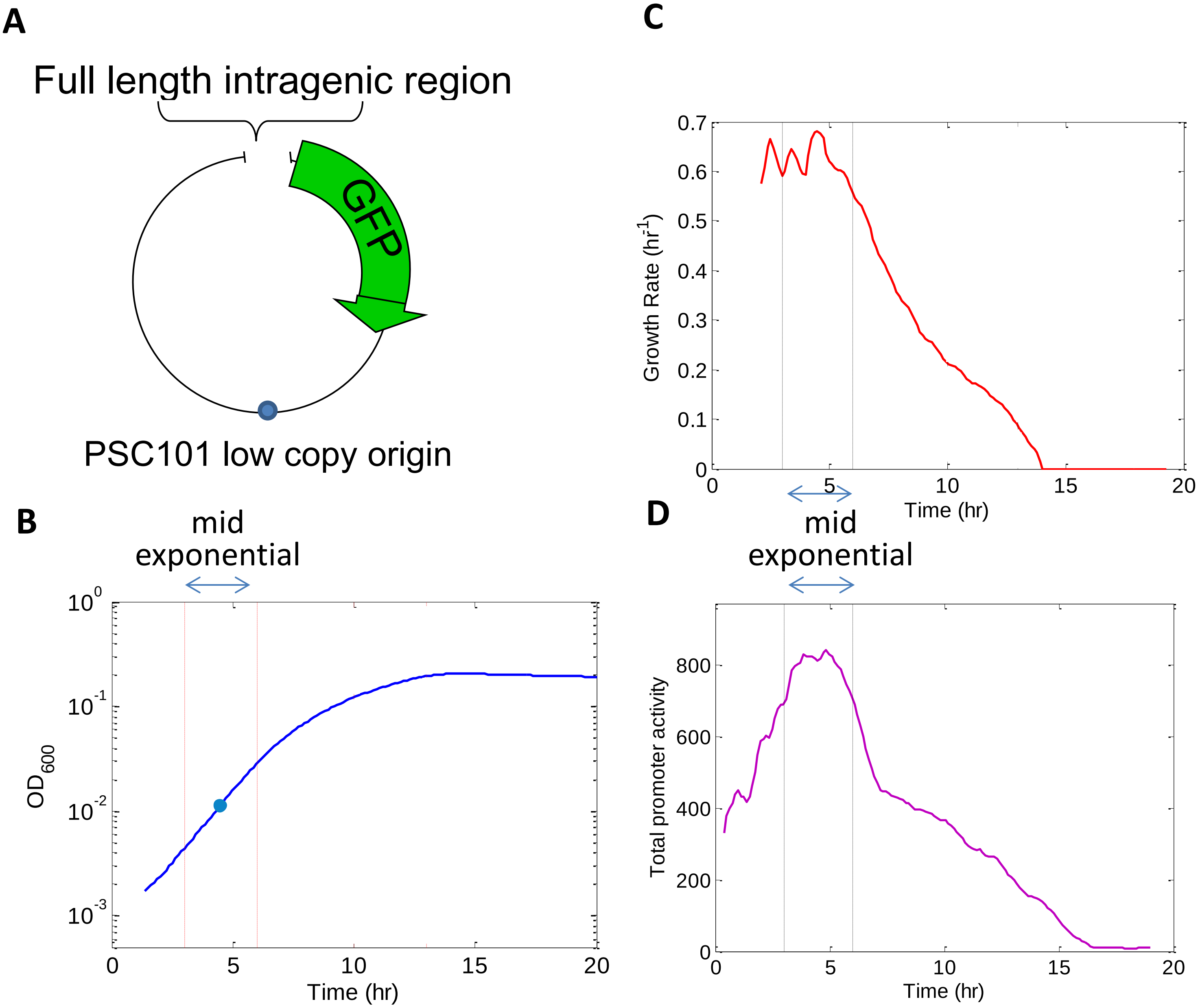
A
Full length intragenic region
GFP
PSC101 low copy origin
B
mid
exponential
10^{0}
10^{-1}
10^{-2}
10^{-3}
OD_{600}
0
5
10
15
20
Time (hr)
C
Growth Rate (hr^{-1})
0.7
0.6
0.5
0.4
0.3
0.2
0.1
0
0
5
10
15
20
Time (hr)
D
mid
exponential
Total promoter activity
800
600
400
200
0
0
5
10
15
20
Time (hr)

Supplementary figure 14. Measurement system for promoter activity in *E. coli* using a library of reporter strains. **(A)** Reporter low-copy plasmid has a full length intragenic region from E coli MG1655 driving the rapidly folding, non-toxic green fluorescent protein variant gfpmut2. Altogether, 1800 strains each corresponding to a different promoter were grown in 96-well plates in 37°C shaker incubator, and robotically moved every 8 min to a multi-well fluorometer for measuring GFP fluorescence and optical density over 24h of growth. **(B)** OD curves match to within 1% median relative error. Exponential phase is defined as point of maximal growth rate. **(C)** Shown is mid-exponential on the growth rate curve **(D)** Promoter activity is $PA = \frac{\partial GFP}{\partial dt}/OD$ at mid-exponential averaged over a 3h time window.

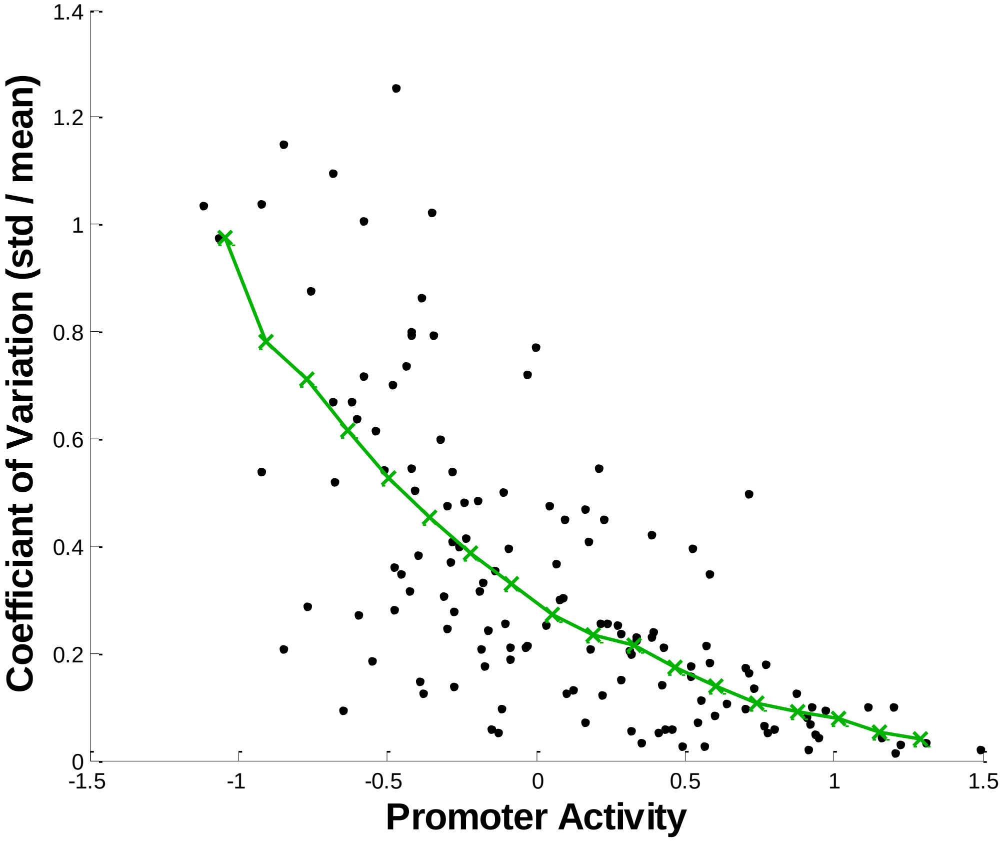

Supplementary figure 15. Estimating the experimental variability of *E. coli* promoter activities. For 3 measured replicates in glucose, shown is the coefficient of variation (CV, standard-deviation divided by the mean, y-axis) of its activity against its mean activity. The measurements were grouped by their promoter activity into 13 equally-spaced bins (in logarithmic scale, green X marks). Also shown (green curve) is a linear interpolation of the CV of each promoter activity using the CVs of four neighboring bins. We used this linear interpolation as an estimate for the relative error of promoters that were not measured in replicates.

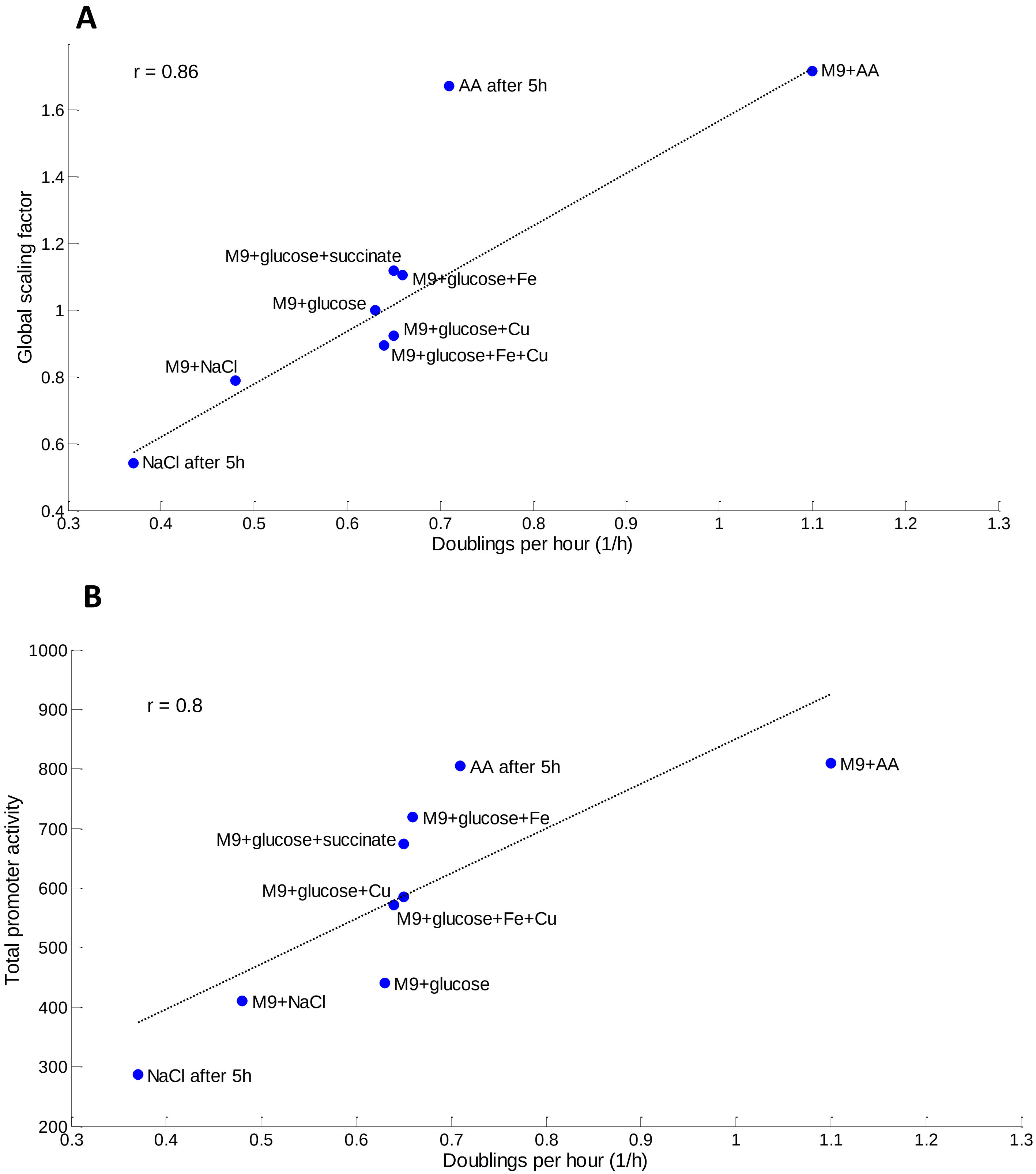

Supplementary figure 16. Global scaling factors and total promoter activity in *E. coli* are correlated to growth rate. For each growth condition, shown is the growth rate (x-axis) and (**A**) global scaling factor or (**B**) total promoter activity (y-axis). The scaling factor for M9+glucose was arbitrarily set to 1.

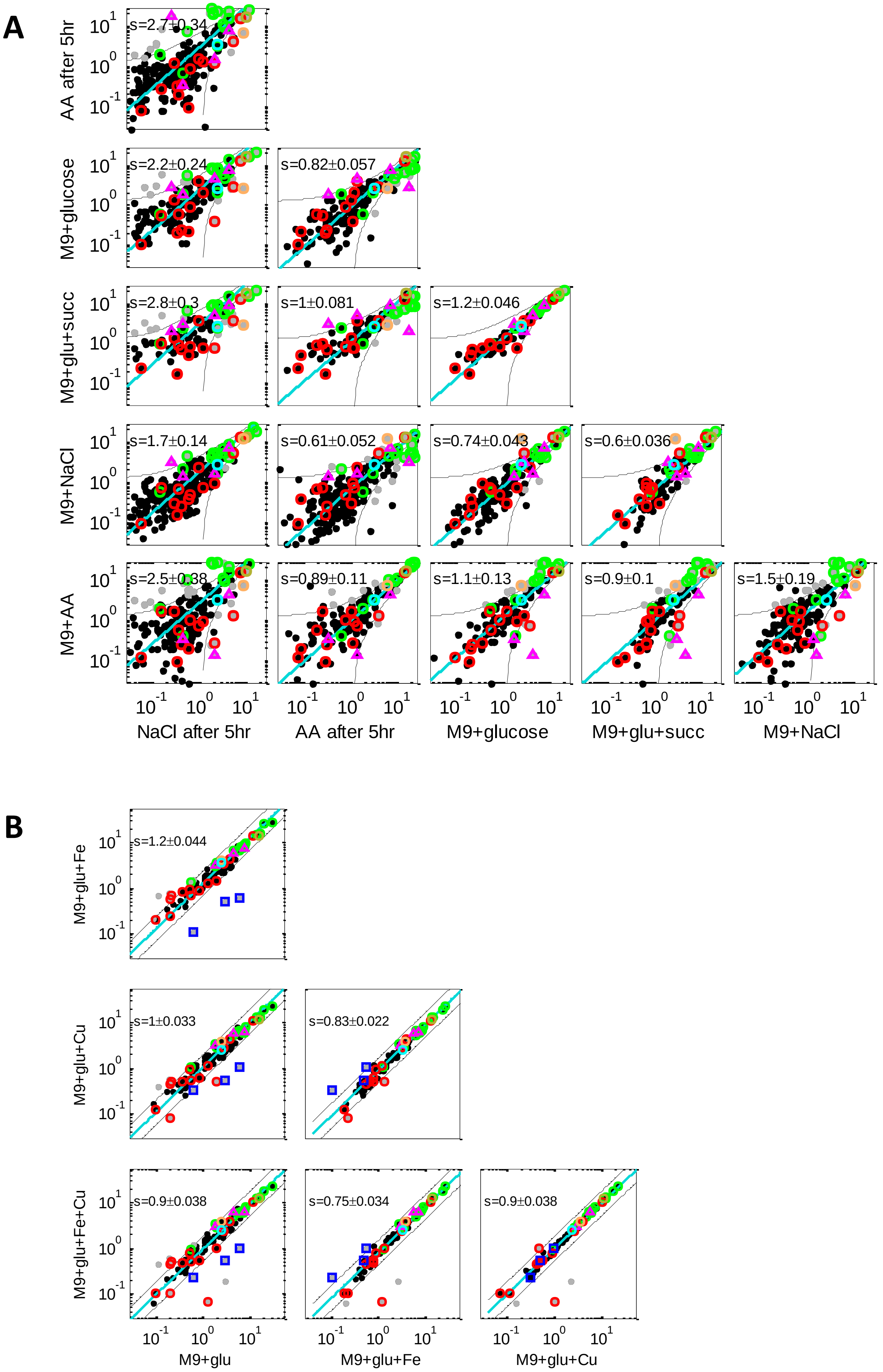

A
AA after 5hr
M9+glucose
M9+glu+succ
M9+NaCl
M9+AA
NaCl after 5hr
AA after 5hr
M9+glucose
M9+glu+succ
M9+NaCl
s=2.7±0.34
s=2.2±0.24
s=0.82±0.057
s=2.8±0.3
s=1±0.081
s=1.2±0.046
s=1.7±0.14
s=0.61±0.052
s=0.74±0.043
s=0.6±0.036
s=2.5±0.38
s=0.89±0.11
s=1.1±0.13
s=0.9±0.1
s=1.5±0.19
B
M9+glu+Fe
M9+glu+Cu
M9+glu+Fe+Cu
M9+glu
M9+glu+Fe
M9+glu+Cu
s=1.2±0.044
s=1±0.033
s=0.83±0.022
s=0.9±0.038
s=0.75±0.034
s=0.9±0.038

Supplementary figure 17. Promoter activity of most *E. coli* promoters in two conditions scale proportionally. **(A)** Promoter activity in 15 pairs of six conditions are compared. Black lines represent 3 standard deviations around the best fit to $P_Y = s \cdot P_X$, (cyan line). Where P_X and P_Y are the promoter activities in the two conditions, and s is the scaling factor. Promoters are colored black or gray depending on whether they fall within or outside the black lines, respectively. Functional groups indicated are ribosomal promoters (green circles), stress proteins (red circles), amino-acid biosynthesis (magenta), ompC (peach), ompA (olive), ferric proteins (blue squares). Indicated is the scale factor s (errors are 95% confidence for regression of log data). **(B)** Same as (A), zooming in on four conditions, differing only by the metals added to the media (iron, copper or both).

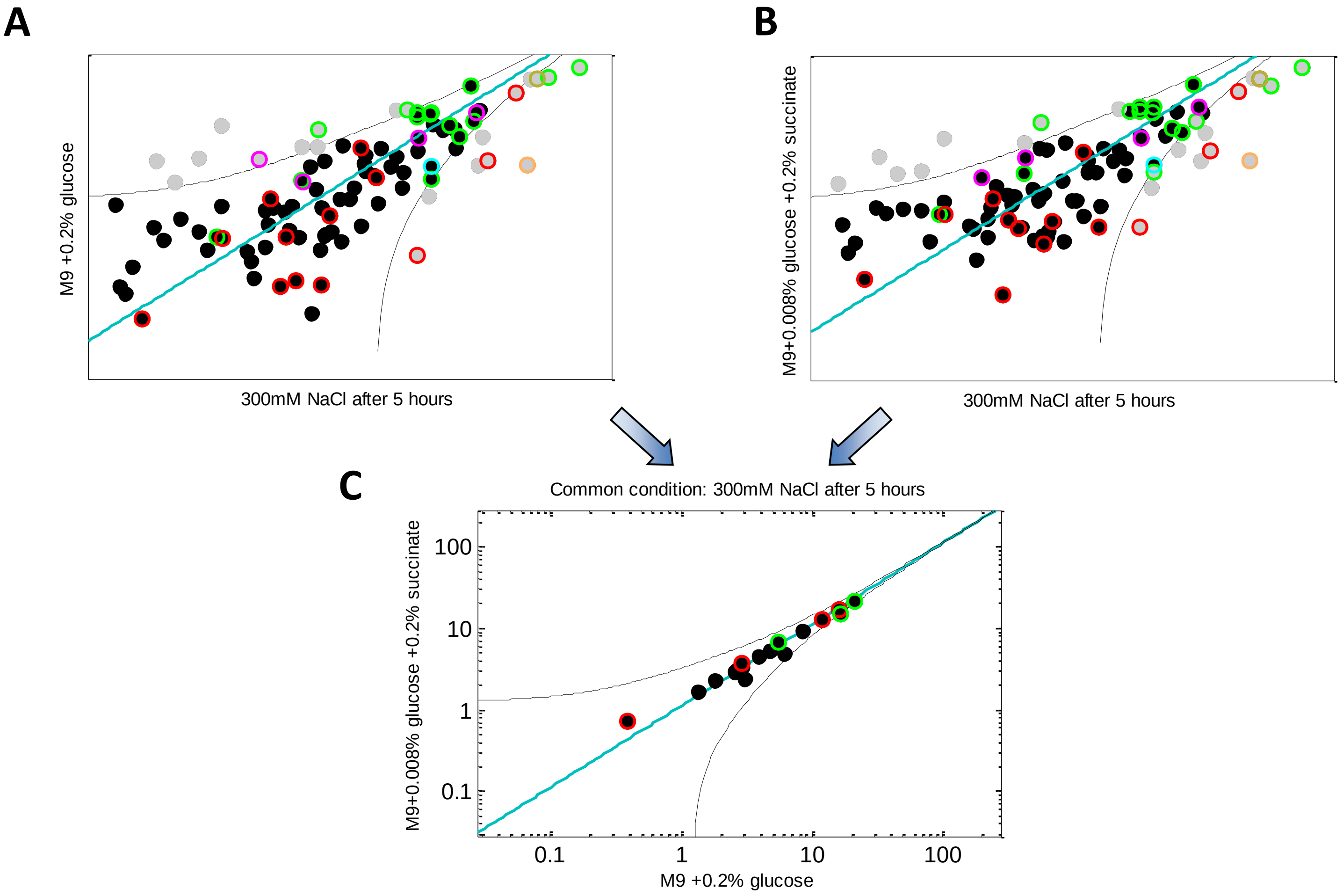

Supplementary figure 18. Condition-specific promoters in *E. coli* preserve proportionality across conditions in which they are activated. **(A-B)** Same as Fig. S13, comparing promoter activities in 300mM NaCl (x-axis) and glucose (y-axis, A) or glucose+succinate (B). **(C)** Shown is a comparison of shared condition-specific promoters (blue dots) from (A) and (B) between glucose (x-axis) and glucose+succinate (y-axis). Promoters display proportional activities, as indicated by their alignment to a straight line. The scaling factor for this subset of promoters (black line) is nearly identical to the global scaling factor (red line) with which the majority of promoters scale between these two conditions.

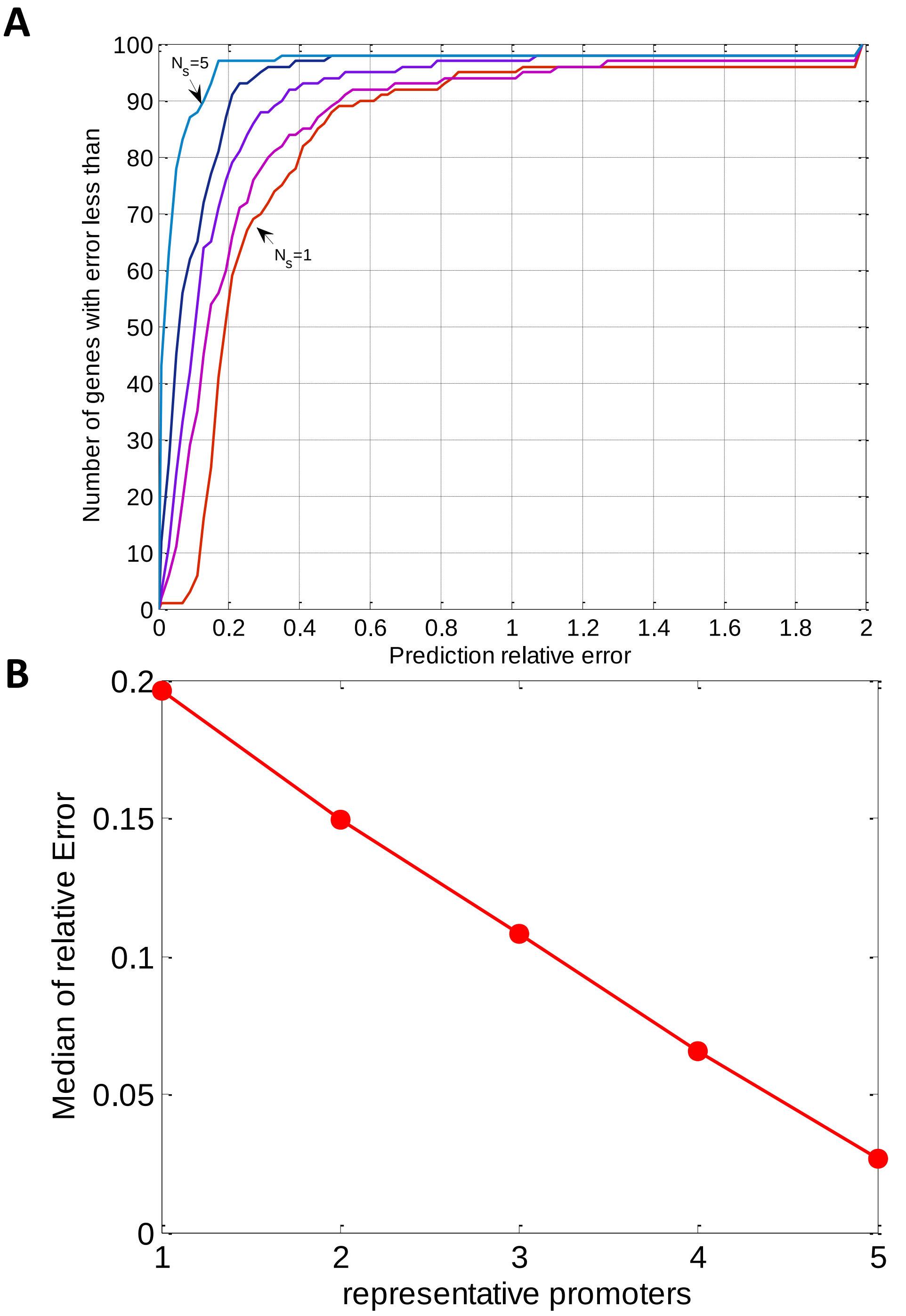

Supplementary figure 19. The promoter activity of the 100 promoters expressed in all conditions is well predicted by a small number of representative promoters. Using all promoters in a set of conditions, we found weights for each promoter such that its expression is given as accurately as possible by the weighted sum of the representative source genes. We numerically searched for the k promoter that best explain the rest. **(A)** Cumulative distribution of prediction error for predictions using N=1 to N=5 representative promoters. **(B)** Mean relative error of prediction (y-axis) using various number of promoters (x-axis). The promoters used for prediction are: rpsP, rrnH, asd, trpL, ompC.

References:

Ashburner M, Ball CA, Blake JA, Botstein D, Butler H, Cherry JM, Davis AP, Dolinski K, Dwight SS, Eppig JT, Harris MA, Hill DP, Issel-Tarver L, Kasarskis A, Lewis S, Matese JC, Richardson JE, Ringwald M, Rubin GM & Sherlock G (2000) Gene ontology: tool for the unification of biology. The Gene Ontology Consortium. *Nature genetics* **25:** 25–9 Available at: http://www.pubmedcentral.nih.gov/articlerender.fcgi?artid=3037419&tool=pmcentrez&rendertype=abstract [Accessed July 19, 2011]

Badis G, Chan ET, van Bakel H, Pena-Castillo L, Tillo D, Tsui K, Carlson CD, Gossett AJ, Hasinoff MJ, Warren CL, Gebbia M, Talukder S, Yang A, Mnaimneh S, Terterov D, Coburn D, Li Yeo A, Yeo ZX, Clarke ND, Lieb JD, *et al* (2008) A library of yeast transcription factor motifs reveals a widespread function for Rsc3 in targeting nucleosome exclusion at promoters. *Molecular cell* **32:** 878–87 Available at: http://www.pubmedcentral.nih.gov/articlerender.fcgi?artid=2743730&tool=pmcentrez&rendertype=abstract [Accessed July 20, 2011]

Bakel H van & Holstege and FCP (2008a) A Tutorial for DNA Microarray Expression Profiling. *Cell*

Bakel H Van & Holstege FCP (2008b) A Tutorial for DNA Microarray Expression Profiling. *Cell* **Evaluating:** 22–28 Available at: http://download.cell.com/images/edimages/etbr/VANBAKEL.PDF

Balázsi G & Oltvai ZN (2007) A pitfall in series of microarrays: the position of probes affects the cross-correlation of gene expression profiles. *Methods in molecular biology (Clifton, N.J.)* **377:** 153–62 Available at: http://www.ncbi.nlm.nih.gov/pubmed/17634615 [Accessed August 14, 2012]

Bammler T, Beyer RP, Bhattacharya S, Boorman GA, Boyles A, Bradford BU, Bumgarner RE, Bushel PR, Chaturvedi K, Choi D, Cunningham ML, Deng S, Dressman HK, Fannin RD, Farin FM, Freedman JH, Fry RC, Harper A, Humble MC, Hurban P, *et al* (2005) Standardizing global gene expression analysis between laboratories and across platforms. *Nature methods* **2:** 351–6 Available at: http://www.ncbi.nlm.nih.gov/pubmed/15846362 [Accessed August 14, 2012]

Benjamini Y & Hochberg Y (1995) Controlling the false discovery rate: a practical and powerful approach to multiple testing. *Journal of the Royal Statistical Society Series B Methodological* **57:** 289–300 Available at: http://www.mendeley.com/catalog/controlling-false-discovery-rate-practical-powerful-approach-multiple-testing-6/ [Accessed March 15, 2013]

Brauer MJ, Huttenhower C, Airoldi EM, Rosenstein R, Matese JC, Gresham D, Boer VM, Troyanskaya OG & Botstein D (2008) Coordination of growth rate, cell cycle, stress response, and metabolic activity in yeast. *Molecular biology of the cell* **19:** 352–67 Available at: http://www.molbiolcell.org/cgi/content/abstract/19/1/352 [Accessed July 21, 2011]

Breker M, Gymrek M & Schuldiner M (2013) A novel single-cell screening platform reveals proteome plasticity during yeast stress responses. *The Journal of cell biology* **200:** 839–50 Available at: http://www.ncbi.nlm.nih.gov/pubmed/23509072 [Accessed August 6, 2013]

Bremer H & Dennis P (1987) Modulation of chemical composition and other parameters of the cell by growth rate. *Escherichia coli and Salmonella …* Available at: http://ctbp.ucsd.edu/qbio/beemer96.pdf [Accessed November 7, 2011]

Broach JR (2012) Nutritional control of growth and development in yeast. *Genetics* **192:** 73–105 Available at: http://www.genetics.org/content/192/1/73.long [Accessed August 7, 2013]

Bronstein I, Fortin J, Stanley PE, Stewart GS & Kricka LJ (1994) Chemiluminescent and bioluminescent reporter gene assays. *Analytical biochemistry* **219:** 169–81 Available at: http://dx.doi.org/10.1006/abio.1994.1254 [Accessed August 14, 2012]

Castrillo JI, Zeef LA, Hoyle DC, Zhang N, Hayes A, Gardner DCJ, Cornell MJ, Petty J, Hakes L, Wardleworth L, Rash B, Brown M, Dunn WB, Broadhurst D, O'Donoghue K, Hester SS, Dunkley TPJ, Hart SR, Swainston N, Li P, *et al* (2007) Growth control of the eukaryote cell: a systems biology study in yeast. *Journal of biology* **6:** 4 Available at: http://www.pubmedcentral.nih.gov/articlerender.fcgi?artid=2373899&tool=pmcentrez&rendertype=abstract [Accessed November 7, 2011]

Chechik G, Oh E, Rando O, Weissman J, Regev A & Koller D (2008) Activity motifs reveal principles of timing in transcriptional control of the yeast metabolic network. *Nature biotechnology* **26:** 1251–9 Available at: http://www.pubmedcentral.nih.gov/articlerender.fcgi?artid=2651818&tool=pmcentrez&rendertype=abstract [Accessed July 13, 2012]

Churchill GA (2002) Fundamentals of experimental design for cDNA microarrays. *Nature genetics* **32 Suppl:** 490–5 Available at: http://www.ncbi.nlm.nih.gov/pubmed/12454643 [Accessed July 17, 2012]

Cormack BP, Valdivia RH & Falkow S (1996) FACS-optimized mutants of the green fluorescent protein (GFP). *Gene* **173:** 33–8 Available at: http://www.ncbi.nlm.nih.gov/pubmed/8707053 [Accessed August 22, 2012]

Costenoble R, Picotti P, Reiter L, Stallmach R, Heinemann M, Sauer U & Aebersold R (2011) Comprehensive quantitative analysis of central carbon and amino-acid metabolism in Saccharomyces cerevisiae under multiple conditions by targeted proteomics. *Molecular systems biology* **7:** 464 Available at: http://www.pubmedcentral.nih.gov/articlerender.fcgi?artid=3063691&tool=pmcentrez&rendertype=abstract [Accessed July 19, 2012]

Cox RS, Surette MG & Elowitz MB (2007) Programming gene expression with combinatorial promoters. *Molecular systems biology* **3:** 145 Available at: http://www.pubmedcentral.nih.gov/articlerender.fcgi?artid=2132448&tool=pmcentrez&rendertype=abstract [Accessed July 15, 2012]

Eden E, Navon R, Steinfeld I, Lipson D & Yakhini Z (2009) GOrilla: a tool for discovery and visualization of enriched GO terms in ranked gene lists. *BMC bioinformatics* **10:** 48 Available at: http://www.pubmedcentral.nih.gov/articlerender.fcgi?artid=2644678&tool=pmcentrez&rendertype=abstract [Accessed June 10, 2011]

Ehrenberg M & Kurland CG (1984) Costs of accuracy determined by a maximal growth rate constraint. *Quarterly reviews of biophysics* **17:** 45–82 Available at: http://www.ncbi.nlm.nih.gov/pubmed/6484121 [Accessed August 15, 2012]

Fazio A & Jewett M (2008) Transcription factor control of growth rate dependent genes in Saccharomyces cerevisiae: A three factor design. *BMC …* Available at: http://www.biomedcentral.com/1471-2164/9/341 [Accessed November 7, 2011]

Frantz S (2005) An array of problems. *Nature reviews. Drug discovery* **4:** 362–3 Available at: http://www.ncbi.nlm.nih.gov/pubmed/15902768 [Accessed August 14, 2012]

Gasch AP, Spellman PT, Kao CM, Carmel-Harel O, Eisen MB, Storz G, Botstein D & Brown PO (2000) Genomic expression programs in the response of yeast cells to environmental changes. *Molecular biology of the cell* **11:** 4241–57 Available at: http://www.pubmedcentral.nih.gov/articlerender.fcgi?artid=15070&tool=pmcentrez&rendertype=abstract [Accessed July 19, 2011]

Gertz J, Siggia ED & Cohen BA (2009) Analysis of combinatorial cis-regulation in synthetic and genomic promoters. *Nature* **457:** 215–8 Available at: http://www.pubmedcentral.nih.gov/articlerender.fcgi?artid=2677908&tool=pmcentrez&rendertype=abstract [Accessed August 11, 2012]

Ghaemmaghami S, Huh W-K, Bower K, Howson RW, Belle A, Dephoure N, O'Shea EK & Weissman JS (2003) Global analysis of protein expression in yeast. *Nature* **425:** 737–41 Available at: http://www.ncbi.nlm.nih.gov/pubmed/14562106 [Accessed July 17, 2012]

De Godoy LMF, Olsen J V, Cox J, Nielsen ML, Hubner NC, Fröhlich F, Walther TC & Mann M (2008) Comprehensive mass-spectrometry-based proteome quantification of haploid versus diploid yeast. *Nature* **455:** 1251–4 Available at: http://www.ncbi.nlm.nih.gov/pubmed/18820680 [Accessed July 17, 2012]

Holstege FC, Jennings EG, Wyrick JJ, Lee TI, Hengartner CJ, Green MR, Golub TR, Lander ES & Young RA (1998) Dissecting the regulatory circuitry of a eukaryotic genome. *Cell* **95:** 717–28 Available at: http://www.ncbi.nlm.nih.gov/pubmed/9845373 [Accessed August 14, 2012]

Houser JR, Ford E, Chatterjea SM, Maleri S, Elston TC & Errede B (2012) An improved short-lived fluorescent protein transcriptional reporter for Saccharomyces cerevisiae. *Yeast (Chichester, England)* **29:** 519–30 Available at: http://www.ncbi.nlm.nih.gov/pubmed/23172645 [Accessed August 4, 2013]

Hughes JD, Estep PW, Tavazoie S & Church GM (2000) Computational identification of cis-regulatory elements associated with groups of functionally related genes in Saccharomyces cerevisiae. *Journal of molecular biology* **296:** 1205–14 Available at: http://www.ncbi.nlm.nih.gov/pubmed/10698627 [Accessed August 4, 2013]

Huisinga KL & Pugh BF (2004) A genome-wide housekeeping role for TFIID and a highly regulated stress-related role for SAGA in Saccharomyces cerevisiae. *Molecular cell* **13:** 573–85 Available at: http://www.ncbi.nlm.nih.gov/pubmed/14992726 [Accessed March 13, 2013]

John L. Ingraham, Ole Maaløe FCN (1983) Growth of the bacterial cell . Sinauer Assoc., Sunderland, MA

Kalir S, McClure J, Pabbaraju K, Southward C, Ronen M, Leibler S, Surette MG & Alon U (2001) Ordering genes in a flagella pathway by analysis of expression kinetics from living bacteria. *Science (New York, N.Y.)* **292:** 2080–3 Available at: http://www.ncbi.nlm.nih.gov/pubmed/11408658 [Accessed July 15, 2012]

Kaplan S, Bren A, Zaslaver A, Dekel E & Alon U (2008) Diverse two-dimensional input functions control bacterial sugar genes. *Molecular cell* **29:** 786–92 Available at: http://www.pubmedcentral.nih.gov/articlerender.fcgi?artid=2366073&tool=pmcentrez&rendertype=abstract [Accessed July 15, 2012]

Keseler IM, Collado-Vides J, Santos-Zavaleta A, Peralta-Gil M, Gama-Castro S, Muñiz-Rascado L, Bonavides-Martinez C, Paley S, Krummenacker M, Altman T, Kaipa P, Spaulding A, Pacheco J, Latendresse M, Fulcher C, Sarker M, Shearer AG, Mackie A, Paulsen I, Gunsalus RP, *et al* (2011) EcoCyc: a comprehensive database of Escherichia coli biology. *Nucleic acids research* **39:** D583–90 Available at: http://www.pubmedcentral.nih.gov/articlerender.fcgi?artid=3013716&tool=pmcentrez&rendertype=abstract [Accessed July 13, 2012]

Klumpp S & Hwa T (2008) Growth-rate-dependent partitioning of RNA polymerases in bacteria. *Proceedings of the National Academy of Sciences of the United States of America* **105:** 20245–50 Available at: http://www.pnas.org/content/105/51/20245.abstract [Accessed September 11, 2013]

Klumpp S, Zhang Z & Hwa T (2009) Growth rate-dependent global effects on gene expression in bacteria. *Cell* **139:** 1366–75 Available at: http://dx.doi.org/10.1016/j.cell.2009.12.001 [Accessed July 15, 2011]

Koch AL (1988) Why can't a cell grow infinitely fast? *Canadian journal of microbiology* **34:** 421–6 Available at: http://www.ncbi.nlm.nih.gov/pubmed/2460206 [Accessed August 15, 2012]

Lai L-C, Kosorukoff AL, Burke P V & Kwast KE (2005) Dynamical remodeling of the transcriptome during short-term anaerobiosis in Saccharomyces cerevisiae: differential response and role of Msn2 and/or Msn4 and other factors in galactose and glucose media. *Molecular and cellular biology* **25:** 4075–91 Available at:

http://www.pubmedcentral.nih.gov/articlerender.fcgi?artid=1087712&tool=pmcentrez&rendertype=abstract [Accessed August 14, 2012]

Levy S & Barkai N (2009) Coordination of gene expression with growth rate: a feedback or a feed-forward strategy? *FEBS letters* **583:** 3974–8 Available at: http://www.febsletters.org/article/S0014-5793(09)00863-1/abstract [Accessed July 18, 2011]

Ligr M, Siddharthan R, Cross FR & Siggia ED (2006) Gene expression from random libraries of yeast promoters. *Genetics* **172:** 2113–22 Available at: http://www.pubmedcentral.nih.gov/articlerender.fcgi?artid=1456374&tool=pmcentrez&rendertype=abstract [Accessed August 14, 2012]

Lipson D, Raz T, Kieu A, Jones DR, Giladi E, Thayer E, Thompson JF, Letovsky S, Milos P & Causey M (2009) Quantification of the yeast transcriptome by single-molecule sequencing. *Nature biotechnology* **27:** 652–8 Available at: http://dx.doi.org/10.1038/nbt.1551 [Accessed July 13, 2012]

Maaloe O (1969) Shaechter69.pdf. In *Developmental Biology* pp 33–58. Elsevier

Magnusson LU, Farewell A & Nyström T (2005) ppGpp: a global regulator in Escherichia coli. *Trends in microbiology* **13:** 236–42 Available at: http://www.ncbi.nlm.nih.gov/pubmed/15866041 [Accessed August 17, 2013]

Marshall E (2004) Getting the noise out of gene arrays. *Science (New York, N.Y.)* **306:** 630–1 Available at: http://www.ncbi.nlm.nih.gov/pubmed/15499004 [Accessed August 14, 2012]

Mateus C & Avery S V. (2000) Destabilized green fluorescent protein for monitoring dynamic changes in yeast gene expression with flow cytometry. *Yeast* **16:** 1313–1323 Available at: http://www.ncbi.nlm.nih.gov/pubmed/11015728 [Accessed July 31, 2013]

Molenaar D, van Berlo R, de Ridder D & Teusink B (2009) Shifts in growth strategies reflect tradeoffs in cellular economics. *Molecular systems biology* **5:** 323 Available at: http://dx.doi.org/10.1038/msb.2009.82 [Accessed March 13, 2012]

Murphy KF, Balázsi G & Collins JJ (2007) Combinatorial promoter design for engineering noisy gene expression. *Proceedings of the National Academy of Sciences of the United States of America* **104:** 12726–31 Available at: http://www.pubmedcentral.nih.gov/articlerender.fcgi?artid=1931564&tool=pmcentrez&rendertype=abstract [Accessed August 14, 2012]

Nagalakshmi U, Wang Z, Waern K, Shou C, Raha D, Gerstein M & Snyder M (2008) The transcriptional landscape of the yeast genome defined by RNA sequencing. *Science (New York, N.Y.)* **320:** 1344–9 Available at: http://www.sciencemag.org/content/320/5881/1344.abstract [Accessed July 13, 2012]

Neidhardt FC (1999) Bacterial Growth: Constant Obsession with dN/dt. *J. Bacteriol.* **181:** 7405–7408 Available at: http://jb.asm.org [Accessed November 7, 2011]

Newman JRS, Ghaemmaghami S, Ihmels J, Breslow DK, Noble M, DeRisi JL & Weissman JS (2006) Single-cell proteomic analysis of S. cerevisiae reveals the architecture of biological noise. *Nature* **441:** 840–6 Available at: http://dx.doi.org/10.1038/nature04785 [Accessed March 9, 2012]

O'Rourke SM & Herskowitz I (2002) A third osmosensing branch in Saccharomyces cerevisiae requires the Msb2 protein and functions in parallel with the Sho1 branch. *Molecular and cellular biology* **22:** 4739–49 Available at: http://www.pubmedcentral.nih.gov/articlerender.fcgi?artid=133928&tool=pmcentrez&rendertype=abstract [Accessed July 18, 2012]

Oshlack A & Wakefield MJ (2009) Transcript length bias in RNA-seq data confounds systems biology. *Biology direct* **4:** 14 Available at: http://www.pubmedcentral.nih.gov/articlerender.fcgi?artid=2678084&tool=pmcentrez&rendertype=abstract [Accessed July 17, 2012]

Pe'er D, Regev A & Tanay A (2002) Minreg: inferring an active regulator set. *Bioinformatics (Oxford, England)* **18 Suppl 1:** S258–67 Available at: http://www.ncbi.nlm.nih.gov/pubmed/12169555 [Accessed August 15, 2012]

Pedersen S, Bloch PL, Reeh S & Neidhardt FC (1978) Patterns of protein synthesis in E. coli: a catalog of the amount of 140 individual proteins at different growth rates. *Cell* **14:** 179–90 Available at: http://www.ncbi.nlm.nih.gov/pubmed/352533 [Accessed September 10, 2012]

Raveh-Sadka T, Levo M, Shabi U, Shany B, Keren L, Lotan-Pompan M, Zeevi D, Sharon E, Weinberger A & Segal E (2012) Manipulating nucleosome disfavoring sequences allows fine-tune regulation of gene expression in yeast. *Nature genetics* **44:** 743–50 Available at: http://dx.doi.org/10.1038/ng.2305 [Accessed July 13, 2012]

Regenberg B, Grotkjaer T, Winther O, Fausbøll A, Akesson M, Bro C, Hansen LK, Brunak S & Nielsen J (2006) Growth-rate regulated genes have profound impact on interpretation of transcriptome profiling in Saccharomyces cerevisiae. *Genome biology* **7:** R107 Available at: http://www.pubmedcentral.nih.gov/articlerender.fcgi?artid=1794586&tool=pmcentrez&rendertype=abstract [Accessed August 4, 2011]

Reményi A, Schöler HR & Wilmanns M (2004) Combinatorial control of gene expression. *Nature structural & molecular biology* **11:** 812–5 Available at: http://dx.doi.org/10.1038/nsmb820 [Accessed October 30, 2012]

Saccharomyces Genome Database Available at: http://www.yeastgenome.org/

Schaechter M (1958) Dependency on medium and temperature of cell size and chemical composition during balanced growth of Salmonella typhimurium. *Journal of general …* **19:**

592–606 Available at: http://mic.sgmjournals.org/content/19/3/592.short [Accessed November 7, 2011]

Scott M, Gunderson CW, Mateescu EM, Zhang Z & Hwa T (2010) Interdependence of cell growth and gene expression: origins and consequences. *Science (New York, N.Y.)* **330:** 1099–102 Available at: http://www.ncbi.nlm.nih.gov/pubmed/21097934 [Accessed March 13, 2012]

Segal E, Shapira M, Regev A, Pe'er D, Botstein D, Koller D & Friedman N (2003) Module networks: identifying regulatory modules and their condition-specific regulators from gene expression data. *Nature genetics* **34:** 166–76 Available at: http://www.ncbi.nlm.nih.gov/pubmed/12740579 [Accessed June 24, 2011]

Shalem O, Dahan O, Levo M, Martinez MR, Furman I, Segal E & Pilpel Y (2008) Transient transcriptional responses to stress are generated by opposing effects of mRNA production and degradation. *Molecular systems biology* **4:** 223 Available at: http://www.pubmedcentral.nih.gov/articlerender.fcgi?artid=2583085&tool=pmcentrez&rendertype=abstract [Accessed July 19, 2012]

Sharon E, Kalma Y, Sharp A, Raveh-Sadka T, Levo M, Zeevi D, Keren L, Yakhini Z, Weinberger A & Segal E (2012) Inferring gene regulatory logic from high-throughput measurements of thousands of systematically designed promoters. *Nature biotechnology* **30:** 521–30 Available at: http://dx.doi.org/10.1038/nbt.2205 [Accessed July 13, 2012]

Stewart-Ornstein J, Weissman JS & El-Samad H (2012) Cellular Noise Regulons Underlie Fluctuations in Saccharomyces cerevisiae. *Molecular cell* **45:** 483–93 Available at: http://www.cell.com/molecular-cell/fulltext/S1097-2765(12)00038-X [Accessed March 3, 2012]

Sun M, Schwalb B, Schulz D, Pirkl N, Etzold S, Larivière L, Maier KC, Seizl M, Tresch A & Cramer P (2012) Comparative dynamic transcriptome analysis (cDTA) reveals mutual feedback between mRNA synthesis and degradation. *Genome research* **22:** 1350–9 Available at: http://genome.cshlp.org/content/early/2012/03/30/gr.130161.111.abstract [Accessed November 11, 2012]

Tang T, François N, Glatigny A, Agier N, Mucchielli M-H, Aggerbeck L & Delacroix H (2007) Expression ratio evaluation in two-colour microarray experiments is significantly improved by correcting image misalignment. *Bioinformatics (Oxford, England)* **23:** 2686–91 Available at: http://www.ncbi.nlm.nih.gov/pubmed/17698492 [Accessed July 14, 2012]

Tirosh I & Barkai N (2008) Two strategies for gene regulation by promoter nucleosomes. *Genome research* **18:** 1084–91 Available at: http://genome.cshlp.org/cgi/content/abstract/18/7/1084 [Accessed July 19, 2011]

Tirosh I, Wong KH, Barkai N & Struhl K (2011) Extensive divergence of yeast stress responses through transitions between induced and constitutive activation. *Proceedings of the National Academy of Sciences of the United States of America* **108:** 16693–8 Available at: http://www.pnas.org/cgi/content/abstract/108/40/16693 [Accessed July 19, 2012]

Wade C, Shea KA, Jensen R V & McAlear MA (2001) EBP2 is a member of the yeast RRB regulon, a transcriptionally coregulated set of genes that are required for ribosome and rRNA biosynthesis. *Molecular and cellular biology* **21:** 8638–50 Available at: http://www.pubmedcentral.nih.gov/articlerender.fcgi?artid=100024&tool=pmcentrez&rendertype=abstract [Accessed August 4, 2013]

Wang M, Weiss M, Simonovic M, Haertinger G, Schrimpf SP, Hengartner MO & von Mering C (2012) PaxDb, a database of protein abundance averages across all three domains of life. *Molecular & cellular proteomics : MCP* **11:** 492–500 Available at: http://www.pubmedcentral.nih.gov/articlerender.fcgi?artid=3412977&tool=pmcentrez&rendertype=abstract [Accessed August 7, 2012]

Yassour M, Kaplan T, Fraser HB, Levin JZ, Pfiffner J, Adiconis X, Schroth G, Luo S, Khrebtukova I, Gnirke A, Nusbaum C, Thompson D-A, Friedman N & Regev A (2009) Ab initio construction of a eukaryotic transcriptome by massively parallel mRNA sequencing. *Proceedings of the National Academy of Sciences of the United States of America* **106:** 3264–9 Available at: http://www.pubmedcentral.nih.gov/articlerender.fcgi?artid=2638735&tool=pmcentrez&rendertype=abstract

You C, Okano H, Hui S, Zhang Z, Kim M, Gunderson CW, Wang Y-P, Lenz P, Yan D & Hwa T (2013) Coordination of bacterial proteome with metabolism by cyclic AMP signalling. *Nature* **advance on:** Available at: http://dx.doi.org/10.1038/nature12446 [Accessed August 7, 2013]

Zaslaver A, Bren A, Ronen M, Itzkovitz S, Kikoin I, Shavit S, Liebermeister W, Surette MG & Alon U (2006) A comprehensive library of fluorescent transcriptional reporters for Escherichia coli. *Nature methods* **3:** 623–8 Available at: http://www.ncbi.nlm.nih.gov/pubmed/16862137 [Accessed July 15, 2012]

Zaslaver A, Kaplan S, Bren A, Jinich A, Mayo A, Dekel E, Alon U & Itzkovitz S (2009) Invariant distribution of promoter activities in Escherichia coli. *PLoS computational biology* **5:** e1000545 Available at: http://dx.plos.org/10.1371/journal.pcbi.1000545 [Accessed July 22, 2011]

Zaslaver A, Mayo AE, Rosenberg R, Bashkin P, Sberro H, Tsalyuk M, Surette MG & Alon U (2004) Just-in-time transcription program in metabolic pathways. *Nature genetics* **36:** 486–91 Available at: http://www.ncbi.nlm.nih.gov/pubmed/15107854 [Accessed July 15, 2012]

Zeevi D, Sharon E, Lotan-Pompan M, Lubling Y, Shipony Z, Raveh-Sadka T, Keren L, Levo M, Weinberger A & Segal E (2011) Compensation for differences in gene copy number among yeast ribosomal proteins is encoded within their promoters. *Genome research* **21:** 2114–28 Available at: http://www.pubmedcentral.nih.gov/articlerender.fcgi?artid=3227101&tool=pmcentrez&rendertype=abstract [Accessed March 26, 2012]

Zhu C, Byers KJRP, McCord RP, Shi Z, Berger MF, Newburger DE, Saulrieta K, Smith Z, Shah M V, Radhakrishnan M, Philippakis AA, Hu Y, De Masi F, Pacek M, Rolfs A, Murthy T, Labaer J &

Bulyk ML (2009) High-resolution DNA-binding specificity analysis of yeast transcription factors. *Genome research* **19:** 556–66 Available at: http://www.pubmedcentral.nih.gov/articlerender.fcgi?artid=2665775&tool=pmcentrez&rendertype=abstract [Accessed June 11, 2011]
